# Fluctuating Growth Rates Link Turnover and Unevenness in Species‐Rich Communities

**DOI:** 10.1111/ele.70333

**Published:** 2026-02-12

**Authors:** Emil Mallmin, Arne Traulsen, Silvia De Monte

**Affiliations:** ^1^ Max Planck Institute for Evolutionary Biology Plön Germany; ^2^ Institut de Biologie de l’ENS (IBENS), Département de Biologie, Ecole Normale Supérieure, CNRS, INSERM Université PSL Paris France

**Keywords:** ecological modelling, fluctuating environments, population dynamics, species abundance distributions

## Abstract

The maintenance of diversity, the ‘commonness of rarity’, and compositional turnover are ubiquitous features of species‐rich communities. Through a minimal model, we consider how these features reflect the interplay between environmental stochasticity, intra‐ and interspecific competition, and dispersal. We show that, even if species have the same time‐average fitness, fluctuations tend to drive the community towards ever‐growing unevenness and species extinctions, but self‐limitation and/or dispersal allow species‐rich states to be sustained. Species abundance–distributions vary systematically in a Buffering–Stabilisation parameter plane that describes the relative strength of the underlying ecological processes, and cover different empirically relevant power‐law and unimodal shapes. A model describing the effective dynamics of a focal species relates static abundance distributions with turnover dynamics, also when species have different mean fitness. The model suggests how community statistics and time series of individual species can inform on the relative importance of the ecological processes that structure diversity.

## Graphical abstract

Through a minimal model of ecological community dynamics, we explain how the interplay between environmental stochasticity, intra‐ and interspecific competition and dispersal relates to the maintenance of diversity, the commonness of rarity and compositional turnover. In particular, species abundance–distributions vary systematically in a Buffering–Stabilisation parameter plane, spanning different empirically relevant power‐law and unimodal shapes.

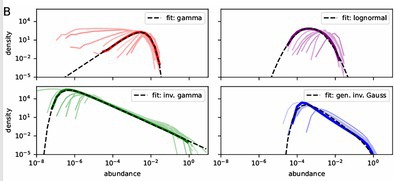

## 1 Introduction

Species‐rich communities—for instance tropical trees, birds, plankton and microbiomes—pose many fundamental ecological questions. In light of the competitive exclusion principle (Hardin 1960; Hutchinson 1961; Levin 1970), how do so many species coexist in environments that, seemingly, offer few axes of niche differentiation? At the same time, coexistence is not equitable: at any given time and place of sampling, the vast majority of species have low abundance in comparison to a few highly abundant species (Preston 1948; Enquist et al. 2019). Species abundance distributions (SADs) quantify the spectrum from dominance to rarity in a community, and universally follow a ‘hollow curve’ shape with high index of unevenness (B. J. McGill 2011). Yet, underlying the relative regularity of SADs is high variability in species' abundances across local samples, and thus in the composition of local communities, for reasons that remains largely unexplained by readily measured environmental factors (Soininen 2014; Ser‐Giacomi et al. 2018; Mutshinda et al. 2016; Rogers et al. 2023).

The precise mechanisms promoting (i) the maintenance of diversity, (ii) the ‘commonness of rarity’ and (iii) pervasive temporal turnover in community composition likely differ between taxonomically and environmentally distant communities. The reality of an ocean‐drifting phytoplankter, extracting what amount of light and nutrients it can before being eaten by a zooplankter, is very different from the tropical tree seedling growing to fill the gap in the canopy opened by a full‐grown rival struck down by lightning (Smetacek 2012). Still, competition, dispersal, and demographic and environmental forms of stochasticity are general processes that affect all species‐rich horizontal communities to varying extents (Vellend 2016; B. J. McGill 2018). The ubiquity of many biodiversity patterns suggests that an explanation is rightly sought in terms of the interactions of a few such high‐level processes.

Classical and recent theoretical developments (Chesson 2000; Barabás et al. 2018; West and Shnerb 2022; MacArthur and Wilson 1967; Hubbell 2001) point towards the maintenance of diversity as resulting from equalising processes, making species more similar in competitive ability (e.g., via trade‐offs in environmental tolerances); stabilising processes, giving a per‐capita competitive advantage to species when rare (e.g., less susceptibility to species‐specific disease); and a balancing of (local) extinctions with (re‐)immigration or speciation. For example, deterministic models of complex communities (notably generalised Lotka‐Volterra (Bunin 2017; Barbier et al. 2018) and consumer‐resource models (Advani et al. 2018; Cui et al. 2021)) show that, under quite general assumptions, species‐rich and stable coexistence equilibria are possible if interactions between species are unstructured and ‘disordered’ (equalising) and sufficiently weak compared to intraspecific competition (stabilising). Alternatively, neutral theory (Hubbell 2001, 2005; Volkov et al. 2003; Vallade and Houchmandzadeh 2003) assumes ecological equivalence of species (perfect equalisation) and weak or no density‐dependence (no stabilisation), leaving demographic stochasticity due to the discreteness of individual birth, death, and dispersal events to drive extinctions that are ultimately balanced by metacommunity processes.

Regarding the commonness of rarity, the stable coexistence regime of disordered competition models produces unrealistically *even* SADs, unless carrying capacities are drawn from an *ad hoc* uneven distribution (Barbier and Arnoldi 2017). In contrast, neutral theory has been celebrated for producing realistic SADs where the shape parameter is given an ecological interpretation as dispersal limitation. But to reliably disambiguate between alternative SAD functional forms in empirical data, for example, lognormal or logseries or a host of alternatives, has proven difficult, and was criticised as a weak test of underlying theories (B. McGill 2003; McGill et al. 2007). Nonetheless, a recent comprehensive analysis (Gao et al. 2025) finds a Poisson‐sampled power‐law with exponential decay at high abundance (‘power bend’ (Pueyo 2006)) to be valid across animal, plant, and microbial communities. The value of the (negative) exponent of the power law section—typically near one for animals and plant communities, and with a median of 1.6 for microbial communities—possibly contains information about underlying processes. For instance, neutral theory predicts an exponent of one, unless generalised to include density‐dependent effects (Ser‐Giacomi et al. 2018); logistic growth of independent species with environmental stochasticity (Engen and Lande 1996), or of interacting species with fast stochastic variation in interaction strength (Suweis et al. 2024), results in a gamma distribution, with exponent strictly less than one, unless species are strongly heterogeneous in their demographic rates (Grilli 2020; Descheemaeker et al. 2021).

Rapid turnover in community composition can be expected to relate to environmental stochasticity—fluctuations in growth rates due to unmodelled variability in resources, abiotic conditions, predation pressure, etc.—rather than demographic stochasticity alone (Nee 2005; Kessler et al. 2015). Indeed, environmental but not demographic stochasticity is consistent with the statistics and timescales of empirical abundance fluctuations (Grilli 2020; Lande et al. 2003). Time‐averaged neutral theory (TAN) (Kalyuzhny et al. 2015; Danino and Shnerb 2018) augments neutral theory with environmental stochasticity, such that species differ in fitness at any given moment in time, but have comparable fitness averaged over a sufficiently long time. Interestingly, disordered competition models, in an unstable interaction regime leading to deterministic chaos, display abundance fluctuations similar to the effect of environmental stochasticity, but also require the buffering effects of a metacommunity to sustain such dynamics (Roy et al. 2020; Pearce et al. 2020; Dalmedigos and Bunin 2020; Mallmin et al. 2024; Arnoulx de Pirey and Bunin 2024; Blumenthal et al. 2024). The slope of the power‐law section of the SAD then depends on the immigration rate and can be larger than one.

Guided by the preceding insights, we investigate what minimal combination of ecological processes might simultaneously account for (i), (ii) and (iii). A time‐averaged neutral model formulated in a Lotka‐Volterra framework provides the starting point (Malcai et al. 2002; Melbinger and Vergassola 2015; van Nes et al. 2024; Kessler and Shnerb 2025). Recently, van Nes et al. (2024) employed such a model to suggest an explanation for the (hyper‐)dominance of a few species in a wide range of community data sets. They draw attention to the ‘stickiness’ effect (called ‘diffusive trapping’ in prior work by Dean and Shnerb (2020)), whereby the scaling of abundance fluctuations biases species that become rare to remain rare. Through an exact mapping to replicator dynamics and to condensation phase transitions in physics, we explain why fluctuating growth rates in fact drive the community rapidly towards unevenness and eventually monodominance, unless compensated by other processes. We demonstrate how immigration (as in parallel work by Kessler and Shnerb (2025)) and self‐regulation allow diversity to be maintained long‐term. Then, we derive the SAD, which is a generalised inverse Gaussian distribution, interpolating between several empirically relevant cases, including power bend (Jørgensen 1982; Sichel 1997). The observed shape varies systematically with two non‐dimensional parameters that we interpret as the effective amount of Buffering and Stabilisation, respectively. Finally, we relax the TAN assumption to find that moderate heterogeneity in species intrinsic growth rates can lead to large differences in the fluctuation statistics of species. We discuss the empirical relevance and generality of our results.

## 2 Model

### 2.1 Community Dynamics

We consider a pool of S species that in a local community of interest have abundances $n_{i}\left(t\right)$ (i=1,2,…,S) at time t. The net growth rate of a species depends on interactions within the community, and the effect of the broader, time‐varying environment; we assume these aspects combine additively. For brevity, we refer to the environmentally determined, density‐independent part of the growth rate as *fitness* ^1^, denoted by $r_{i}\left(t\right)$. Following classical hypotheses, we take interactions to be dominated by competition (direct or apparent), such that heterospecifics compete with strength μ, and conspecifics with strength μ+ε. We refer to the special case ε=0 as *uniform competition*, and call ε the *excess self‐regulation*. Moreover, we consider a small, constant rate of net immigration λ. Denoting the total abundance by $N\left(t\right)=\sum_{j=1}^{S}n_{j}\left(t\right)$, the above assumptions define the growth equation

(1) $${\dot{n}}_{i}\left(t\right)=n_{i}\left(t\right)\left[r_{i}\left(t\right)-\mathrm{\mu N}\left(t\right)-\varepsilon n_{i}\left(t\right)\right]+\lambda .$$

We will also consider a spatially explicit metacommunity version of the model, where M patches of local communities are connected through dispersal at rates $d_{\alpha \beta }$ from patch β to α. For the abundance dynamics of species i in patch α, we then replace λ above by the species' immigration into the patch minus the emmigration to all other patches,

(2) $$\sum_{\beta =1}^{M}\left(d_{\alpha \beta }n_{i,\beta }\left(t\right)-d_{\beta \alpha }n_{i,\alpha }\left(t\right)\right).$$

While the community dynamics encompasses some of the most broadly relevant processes, there are also notable omissions. We do not include demographic stochasticity, but to nonetheless allow for the extinction of rare species we introduce a threshold $n_{\mathrm{ext}}$ below which abundances are set to zero. Furthermore, species coexistence through the storage effect (i.e., noise‐induced stabilisation) (Chesson and Warner 1981; Johnson and Hastings 2022) has been precluded, since the fluctuating fitnesses and competition appear additively in the growth rate. For perspectives on these effects in species‐rich communities, we refer to several recent works (Danino and Shnerb 2018; Kessler and Shnerb 2024; Pande and Shnerb 2022).

### 2.2 Fluctuating Fitnesses

The fluctuating fitnesses $r_{i}\left(t\right)$ represent the net effect of a complex and time‐varying environment that we do not model explicitly. For simplicity, we assume the $r_{i}\left(t\right)$s to be statistically independent between species, and density independent. We take each $r_{i}\left(t\right)$ as a coloured noise with expected value $r_{i}^{*}$, variance $\sigma_{r}^{2}$ and autocorrelation time τ. Unless otherwise indicated, we will assume species are *time‐average neutral*, meaning $r_{i}^{*}=r^{*}$ (Kalyuzhny et al. 2015) (Fitness variance and autocorrelation will be species‐independent throughout.) We let the fitness dynamics follow an Ornstein‐Uhlenbeck process

(3) $$\tau {\dot{r}}_{i}\left(t\right)=-\left(r_{i}\left(t\right)-r_{i}^{*}\right)+\sqrt{2\sigma_{r}^{2}\tau }{\dot{W}}_{i}\left(t\right),$$

where ${\dot{W}}_{i}\left(t\right)$ formally represents white noise. At stationarity, fitnesses follow a normal distribution $N\left(r_{i}^{*},\sigma_{r}^{2}\right)$.

Later, we will identify the parameter combination

(4) $$\gamma ≔2\sigma_{r}^{2}\tau$$

appearing in Equation (3) as the *rate of stochastic exclusion*. We will therefore often specify the noise in terms of $\left(r^{*},\gamma ,\tau \right)$ (which implies the value of $\sigma_{r}$ through Equation (4)). In the *fast environment limit* of τ→0, $\sigma_{r}\to \infty$, while keeping γ constant, one obtains a white noise $r_{i}\left(t\right)=r_{i}^{*}+\sqrt{\gamma }{\dot{W}}_{i}$ (in the Stratonovich stochastic calculus (Pesce et al. 2013)). Sticking to coloured noise has several advantages, however: we do not implicitly assume environmental fluctuation timescales are fast (perhaps reasonable for elephants, but less so for *E. coli* ); $\sigma_{r}$ and τ have a clearer biological interpretation than the noise amplitude γ; and we can ignore subtleties of stochastic calculus convention.

### 2.3 Model Parameters and Simulations

In the fully deterministic, neutral case (ε=0, γ=0, λ=0), the total abundance equilibrates at the *carrying capacity* $K≔r^{*}/\mu$. By rescaling abundances, we can set K=1. We measure time in units of $1/r^{*}$, approximately equal to one generation time, which we set to 1 day for ease of communication and without loss of generality.

All model variables and parameters are summarised in Table S1; parameter values are specified in the figure captions. The numerical implementation of the model is described in Appendix B.

## 3 Results

### 3.1 Randomly Fluctuating Fitnesses Drive Diversity Loss

To establish a baseline for the effect of fitness fluctuations on species coexistence and diversity, we consider the special case of Equation (1) with uniform competition (ε=0) and no immigration $\left(\lambda =0\right)$:

(5) $${\dot{n}}_{i}\left(t\right)=n_{i}\left(t\right)\left[r_{i}\left(t\right)-\mathrm{\mu N}\left(t\right)\right].$$

Below, we study the dynamics of this system, first in simulation and then analytically, with the following main conclusions:

Coexistence in Equation (5) is only transient: communities progress towards pronounced unevenness, and eventually monodominance. This is true even in the absence of an extinction cutoff, in which case it takes progressively longer for the identity of the dominant species to change. The stickiness effect forms part of the explanation (Danino and Shnerb 2018; Kessler and Shnerb 2025): Because the magnitude of abundance fluctuations is proportional to the current abundance, the rarer a species, the larger (and hence more infrequent) the fitness fluctuation needed to escape rarity. The other part can be traced to the growing variance of fitnesses integrated over time, despite the convergence of time‐averaged fitnesses towards $r^{*}$.

A key measure of the effectiveness of stochastic exclusion is the time $t_{c}$ it takes for an initially even community to become composed of a few dominant species. We show that it scales as $\ln\left(S\right)/\gamma$, with γ as in Equation (4). Whether $t_{c}$ is a long or short time on the scale of generations depends primarily on $\gamma /r^{*}$; it is long if relative fluctuations are small ($\sigma_{r}/r^{*}\ll 1)$, or if environmental changes are fast compared to generation time ($\tau \ll 1/r^{*})$. Remarkably, a community of S=10′000 species would only need twice the time to reach few‐species dominance as a 100‐species community, all else being equal. Because $t_{c}∼\gamma^{-1}$, we will refer to γ as the *rate of (stochastic) exclusion*.

### 3.2 Numerical Simulations Reveal Transient Diversity

**FIGURE 1:**
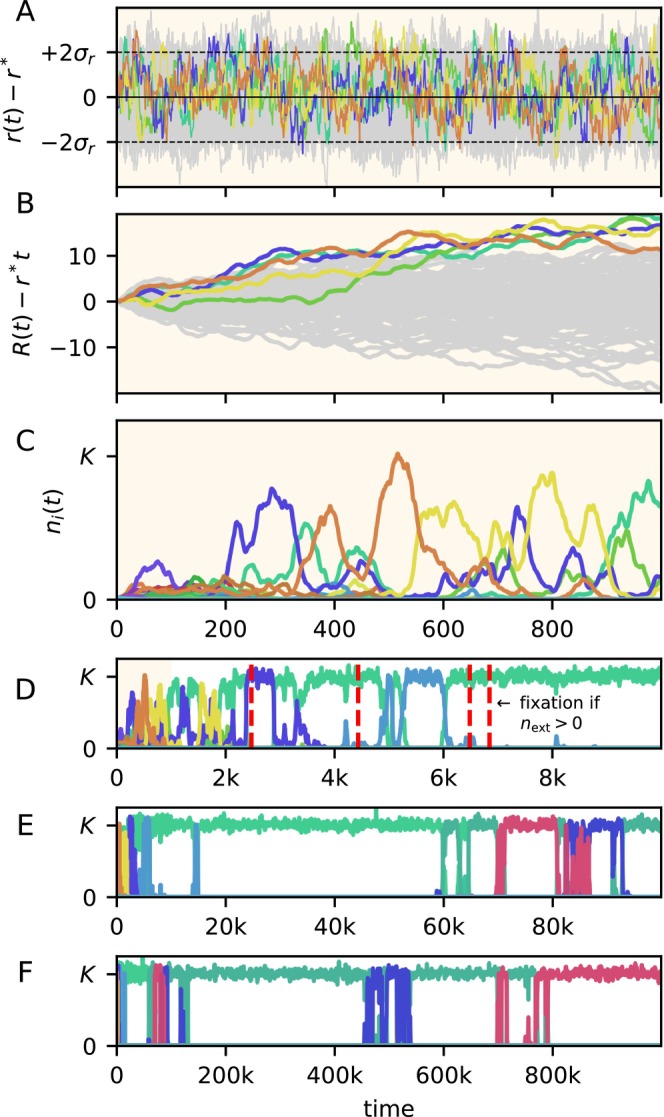
Simulated community dynamics showing progressive unevenness. Single simulation run of Equations (1) and (3), starting from a perfectly even community. Instantaneous fitness (A) and time‐averaged one (B), highlighted in colour for a few highly successful species. The same species are highlighted in the abundance time series (C–F), displayed in increasingly longer time windows. The shown simulation trajectories were generated without an extinction threshold, but the vertical red lines in D indicate when, for the same fitness dynamics as shown, a single species would fixate under different extinction thresholds ($n_{\mathrm{ext}}/K=10^{-3}$, $10^{-6}$, $10^{-9}$, $10^{-12}$). Despite a transient with high species diversity, monodominance is readily attained and is fixed for any positive extinction threshold. Simulation parameters are S=100, K=1, $r^{*}=1$, γ=0.05 τ=10.

To provide intuition on the ecological dynamics of an initially maximally diverse community ($n_{i}\left(0\right)=K/S$, $r_{i}\left(0\right)=r^{*}$), we simulate Equation (5) numerically (Figure 1). We observe that, within a few hundred days, a handful of high‐abundance species stand out (Figure 1C). While it is difficult to judge any species' success by its instantaneous fitness (Figure 1A), the dominant species can be recognised as having the highest time‐integrated fitness since the initial time (Figure 1B). After a few thousand days, the community is dominated by a single species (Figure 1D). As we observe the abundances over long timescales—from years (Figure 1C), to decades (Figure 1D), to centuries (Figure 1E), to millennia (Figure 1F)—the intervals between exchanges of dominance tend to lengthen. Correspondingly, species that are not dominant become increasingly rare, so that, for any positive extinction threshold $n_{\mathrm{ext}}$, the number of extant species progressively decays until only one species remains (varying $n_{\mathrm{ext}}/K$ from $10^{-3}$ to $10^{-12}$ has less than an order of magnitude effect on the timescale of fixation; see Figure 1D). The last surviving species is at no practical risk of stochastic extinction, although, technically, it will vanish eventually.

**FIGURE 2:**
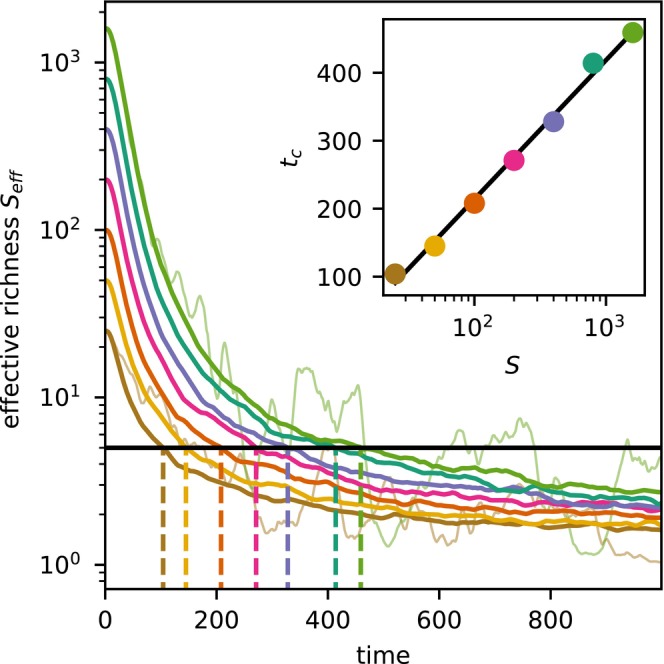
Decay of the effective species richness $S_{\mathrm{eff}}\left(t\right)$ (Equation (6)), starting from initially even community of 25–1600 species. Thick lines show averages over 200 simulations, with the two thin lines illustrating representative single runs for S=25 and 1600. The inset shows the time $t_{c}$ at which the ensemble‐averaged $S_{\mathrm{eff}}\left(t\right)$ has decayed to 5 species, plotted against the initial richness. The initially even community loses its diversity on a timescale of lnS, in agreement with our calculations.

We focus next on the timescale for the community to become highly uneven. As a proxy for the number of dominant species, we measure the *effective richness* by Simpson's reciprocal diversity index:

(6) $$S_{\mathrm{eff}}\left(t\right)≔\frac{1}{\sum_{i}p_{i}^{2}\left(t\right)},$$

where $p_{i}=n_{i}/N$ denote relative abundances. In the initially even community $S_{\mathrm{eff}}\left(0\right)=S$, while $S_{\mathrm{eff}}\to 1$ for large times, signifying monodominance. We measure the time $t_{c}$ at which the effective richness crosses a threshold of a few species. As shown in Figure 2, distributions readily grow uneven also in very large communities, with $t_{c}$ scaling as lnS.

**FIGURE 3:**
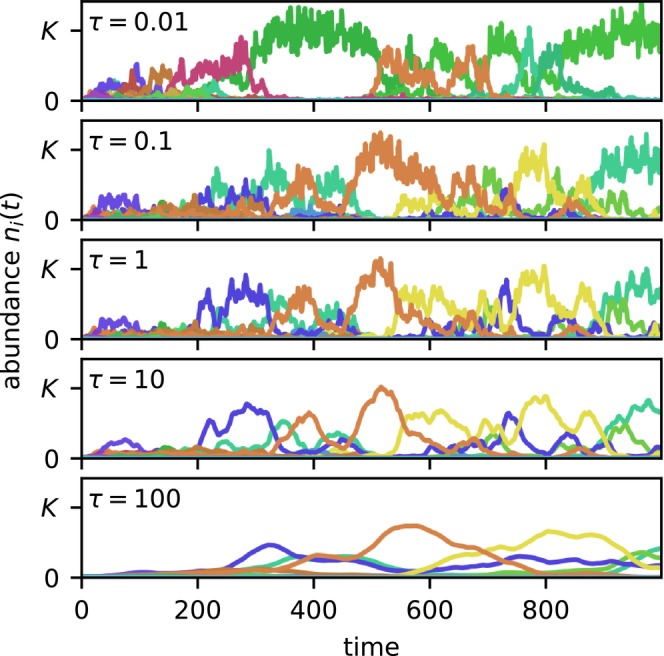
The main trend in community composition is scarcely affected by the fitness autocorrelation time τ if the exclusion rate γ is fixed. The random numbers underlying the simulations are identical for all panels. We have fixed γ=0.05, implying $\sigma_{r}^{2}=0.125\tau$ by Equation (4), and then vary τ between panels.

The critical time $t_{c}$ decreases with the rate of stochastic exclusion γ, as we prove in the next section. Indeed, γ essentially sets the ‘ecological clock’ of the model. As shown in Figure 3, when γ is fixed, the correlation time τ alone has little effect on the main dynamical trends, but controls the extent of rapid fluctuations around them.

### 3.3 A Mapping to the Replicator Equation Explains the Dynamics of Community Unevenness

Despite the large fluctuations of individual species abundances, the total abundance $N\left(t\right)$ fluctuates only moderately. This motivates focussing on the relative abundances $p_{i}=n_{i}/N$, which obey the replicator Equation (57) (Appendix C)

(7) $${\dot{p}}_{i}\left(t\right)=p_{i}\left(t\right)\left[r_{i}\left(t\right)-\rho \left(t\right)\right],$$

where the community‐average fitness $\rho \left(t\right)≔\sum_{j}r_{j}\left(t\right)p_{j}\left(t\right)$. This result follows from Equation (1) regardless of the functional form of $r_{i}\left(t\right)$, and is in fact independent of the uniform competition term $\mathrm{\mu N}\left(t\right)$ or its generalisation to any function that has the same value for all species. On the other hand, the strength of the uniform competition μ constrains the total abundance, whose dynamics

(8) $$\dot{N}\left(t\right)=N\left(t\right)\left[\rho \left(t\right)-\mathrm{\mu N}\left(t\right)\right]$$

is coupled to community composition only through $\rho \left(t\right)$.

Key to understanding the dynamics of species composition are the time‐integrated fitnesses

(9) $$R_{i}\left(t\right)≔\int_{0}^{t}dt^{\prime }\;r_{i}\left(t^{\prime }\right),$$

as appreciated from the formal solution to Equation (7):

(10) $$p_{i}\left(t\right)=\frac{p_{i}\left(0\right)e^{R_{i}\left(t\right)}}{Z\left(t\right)},\;Z\left(t\right)≔\sum_{j=1}^{S}p_{j}\left(0\right)e^{R_{j}\left(t\right)}.$$

A species i becomes dominant when the factor $e^{R_{i}}$ makes up a sizeable fraction of the sum of exponentials, so that the question of dominance is essentially one of extreme value statistics. If the gap between the largest (or largest few) $R_{i}$ and the rest tends to grow in time, then eventually—and, indeed, rather soon due to the exponentiation—the corresponding species will come to dominate. If species differ in their expected fitnesses, the one with larger average fitness eventually wins deterministically (competitive exclusion). In the time‐averaged neutral case with random fitness fluctuations following Equation (3), after a transient of length comparable to τ, the $R_{i}$s diverge at rate γ (see Equation (22) in Appendix A), which thus controls the speed at which community unevenness develops. Also, the aging dynamics observed for $n_{\mathrm{ext}}=0$, where changes in dominance become increasingly rare, is explained by the property of Brownian motions $W_{i}$ to return to the origin in finite time despite the growing variance. In contrast, there is no asymptotic monodominance if fluctuations are perfectly periodical, for example, $r_{i}\left(t\right)=r^{*}+\sqrt{2}\sigma_{r}\cos\left(t/\tau -\phi_{i}\right)$, because the variance among $R_{i}$s remains bounded.

We note that Equation (10) has the form of the Boltzmann distribution from equilibrium statistical physics. Indeed, in Appendix E we show how the ecological model with Gaussian fitness fluctuations can be exactly mapped to the ‘random energy model’ of a spin glass, for which many properties have been calculated in the large‐system limit (Derrida 1980). The spin glass exhibits a condensation phase transition at a critical temperature, which is mathematically analogous to the community unevenness transition at a critical time Equation (38) scaling as

(11) $$t_{c}∼\frac{\ln S}{\gamma },$$

on the assumption that $\tau \ll t_{c}$. This analytical result matches the scaling of $t_{c}$ with lnS that we observed in simulations (Figure 2).

The dynamics of community unevenness can also be understood by looking at a single *focal species*. For a community of two species (Danino and Shnerb 2018), using $p_{2}=1-p_{1}$ in Equation (7),

(12) $${\dot{p}}_{1}\left(t\right)=\Delta r_{1}\left(t\right)p_{1}\left(t\right)\left[1-p_{1}\left(t\right)\right],$$

with $\Delta r_{1}\left(t\right)≔r_{1}\left(t\right)-r_{2}\left(t\right)$, which is independent of any species' abundance. As the relative abundance of the focal species 1 approaches either 0 or 1, the dynamics slows down, keeping the species generally closer to these extremes than at any intermediate value. We show in Appendix D that Equation (12) holds for a focal species also in an S‐species community, given a generalised form of $\Delta r_{1}$. Consider the sub‐community of all species *except* the focal one, and denote by $\rho_{\setminus 1}\left(t\right)$ the mean fitness in this subcommunity (i.e., where relative abundances are normalised only with respect to the S−1 non‐focal species). Then Equation (12) holds for

(13) $$\Delta r_{1}\left(t\right)≔r_{1}\left(t\right)-\rho_{\setminus 1}\left(t\right).$$

Unlike the two‐species case, $\Delta r_{1}$ now has a negative bias: $\rho_{\setminus 1}$ is weighted towards the species with higher abundances, which tend to have higher‐than‐average growth rates. Thus, all species are biased towards rarity, but since relative abundances are normalised—a constraint enforced through correlations between all the $\Delta r_{i}$s—some species will buck the trend and seize a large fraction of the total abundance. We note that similar dynamical aging appears in a deterministic model where $\Delta r_{1}$ encompasses heterogeneous species interactions (Arnoulx de Pirey and Bunin 2023).

### 3.4 Species Loss Is Drastically Slowed by Intraspecific Limitation or Metacommunity Buffering

As we have demonstrated, environmental stochasticity can drive ‘commonness of rarity’ and turnover of composition, but only transiently. Long‐term maintenance of species richness requires local coexistence mechanisms (Barabás et al. 2018; Chesson and Kuang 2008), or extinction–colonisation balance (MacArthur and Wilson 1967; Hubbell 2001). We therefore consider the effects of additional intraspecific limitation or metacommunity dispersal on diversity.

We suppose intraspecific competition exceeds interspecific competition by an amount ε>0:

(14) $${\dot{n}}_{i}\left(t\right)=n_{i}\left(t\right)\left[r_{i}\left(t\right)-\mathrm{\mu N}\left(t\right)-\varepsilon n_{i}\right].$$

**FIGURE 4:**
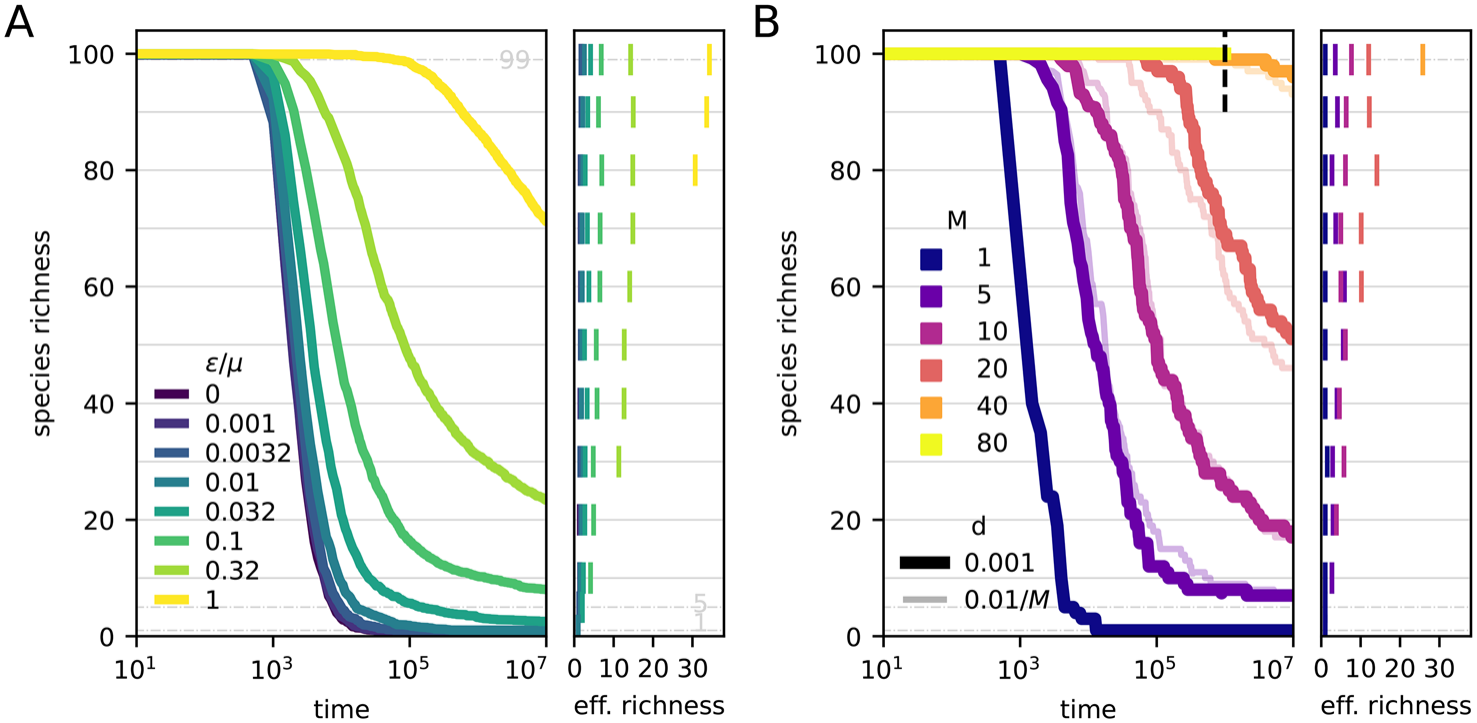
Loss off species richness (large panel) and effective richness Equation (6) (side panel) over time. The effective richnesses are plotted for the times at which the absolute abundance crosses the level values indicated by horizontal grey lines. The average diversity decay over 20 simulation runs is plotted for different values of excess self‐regulation ε (A), and in metapopulations with different number of patches M and migration rates d (B). Other parameters are as given in Figure 1 with $n_{\mathrm{ext}}/K=10^{-12}$.

This introduces negative frequency dependence, such that a species is penalised (favoured) when its relative abundance is above (below) $1/S_{\mathrm{eff}}$ (see Equation (46), Appendix F). In principle, any ε>0 stabilises coexistence (Appendix G), but only in the absence of an extinction threshold. As we allow for extinctions, increasing ε/μ from 0 to 1 increases the timescale of substantial loss of species richness by many orders of magnitude (Figure 4A). The effective species richness remains roughly constant until constrained by the absolute richness, as the rare species headed towards extinction have a marginal effect on the rest of the community. It is therefore reasonable to consider the community as quasi‐stationary on timescales that can indeed be very long even when self‐regulation is weak. Further increasing ε/μ to around $3S\sigma_{r}/r^{*}$ (= 15 with default simulation parameters) would allow essentially all species to coexist deterministically if the fitnesses were suddenly frozen, that is, drawn statically from the stationary distribution (Appendix H); then stochastic exclusion does not occur at all, in practice.

Alternatively, we introduce a metacommunity buffering effect through self‐consistent dispersal among M patches:

(15) $${\dot{n}}_{i,\alpha }=n_{i,\alpha }\left(r_{i,\alpha }-\mu N_{\alpha }\right)+\sum_{\beta =1}^{M}\left(d_{\alpha \beta }n_{i,\beta }-d_{\beta \alpha }n_{i,\alpha }\right).$$

Naturally, the rates of local and regional extinction will depend on the number of patches and the topology of the network, the correlation in environmental conditions between patches, the rates of dispersal, and the extinction threshold; enough factors to make a systematic analysis challenging. In Figure 4B we only consider a fully connected patch network, uncorrelated fitnesses, and vary either the net dispersal rate (d) or the dispersal per patch $\left(d/M\right)$. In either case, every doubling of the number of patches leads to about one more order of magnitude in the time it takes to lose species richness in a given patch. Indeed, related metacommunity models have found species lifetimes to grow exponentially with the number of patches (Roy et al. 2020; Garcia Lorenzana et al. 2024).

### 3.5 A Modified Power‐Law Abundance Distribution Is Maintained by Turnover of Rare and Dominant Species

**FIGURE 5:**
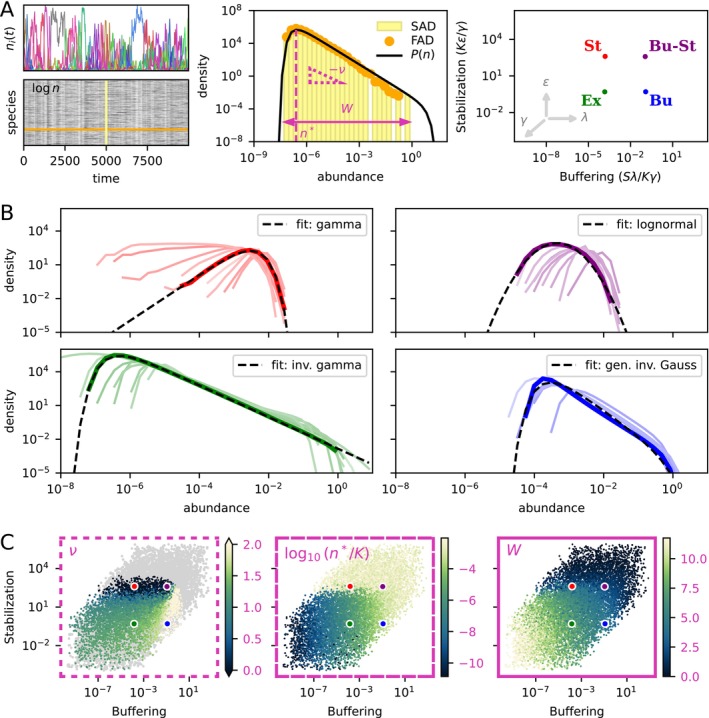
Variationintheshapeofabundancedistributionsacrosssimulatedcommunitieswithvaryingbaseparameters. (A) Left: An example times series with the corresponding abundance matrix (times × species). Middle: Different abundance distributions constructed from the abundance matrix: the ‘snapshot’ species–abundance distribution (SAD, yellow histogram); the frequency–abundance distribution (FAD, orange symbols) for one arbitrary species; the predicted stationary distribution Equation (52) of the focal species model with effective noise statistics measured from the data (solid black line). Three key features of the distributions are highlighted (pink): the number of decades W spanned by the SAD; the modal abundance class $n^{*}$; and the downward slope of the power‐law section, as defined by the formula Equation (18). Right: A reduced parameter space, where each point in the 7‐dimensional base parameter space is mapped to a value of Buffering (horizontal axis) and Stabilisation (vertical axis). The inset arrows show the direction of movement as the indicated parameter (ε, λ, or γ) is changed while all others are held constant. Four points have been marked as references and named according to the dominant process: Ex (Exclusion), St (Stabilisation), Bu (Buffering), Bu‐St (Buffering‐and‐Stabilisation). (B) Four panels corresponding to the four reference points, each showing t10 SADs (from the 10 simulations whose parameters lie closest to the reference points). One SAD has been highlighted (bold) and fitted with a particular distribution (gamma, lognormal, inverse gamma or generalised inverse Gaussian). Figure S2 shows the morphing of one class of shape into another as we move in the Buffering–Stabilisation space. (C) Variation of SAD features across the Buffering–Stabilisation space. Each point represents one simulation. Parameters were sampled (log‐)uniformly to vary over orders of magnitude: $S\in \left[100,1000\right]$, $\log_{10}\gamma \in \left[-4,2\right]$, $\log_{10}\tau \in \left[-2,2\right]$, $\log_{10}\varepsilon \in \left[-2,2\right]$, $\log_{10}\lambda \in \left[-10,-4\right]$. Units are adapted so that K=1 and $r^{*}=1$. In the ν panel, points were excluded (grey) if the focal‐species SAD has a goodness‐of‐fit below 85% (see Figure S1) or the distribution covered less than two decades (W<2). Note that colour scale for the exponent is capped in the range $\left[0,2\right]$, so that all negative values appear in the same colour (dark blue).

Given the radical slowdown of diversity loss achievable by modest amounts of excess self‐regulation or dispersal, we consider in the following the single‐patch dynamics Equations (1) and (3) without extinction cut‐off, which has a true stationary state. We look at two empirically relevant statistics: the abundance distributions displayed by individual species over long stretches of time (frequency–abundance distribution, FAD), or by all species of the community at a snapshot in time (species–abundance distribution, SAD). Their relation is illustrated in Figure 5A. While all species fluctuate in abundance over time, the SAD retains its general shape across snapshots, which appears to be a subsampling of the FAD. Moreover, all species have identical FAD if compared for a sufficiently long time, due to species‐symmetry of the model parameters. Thus, for large, time‐averaged neutral communities, the FAD and SAD essentially coincide.

Seeking to derive the form of the abundance distribution, we consider the dynamics of a focal species, for which the influence of the rest of the community is treated as part of an ‘effective’ fluctuating environment:

(16) $$\dot{n}=n\left(r_{\mathrm{eff}}\left(t\right)-\mathrm{\varepsilon n}\right)+\lambda .$$

We take $r_{\mathrm{eff}}\left(t\right)$ to be an Ornstein‐Uhlenbeck process like Equation (3), but with mean $r_{\mathrm{eff}}$, variance $\sigma_{r_{\mathrm{eff}}}^{2}$, and autocorrelation time $\tau_{\mathrm{eff}}$ (from which we define $\gamma_{\mathrm{eff}}=2\sigma_{r_{\mathrm{eff}}}^{2}\tau_{\mathrm{eff}}$, as before). These statistics are tuned to approximate those of $r_{i}\left(t\right)-\mathrm{\mu N}\left(t\right)$ (see Appendix G). In the fast‐environment limit, the stationary distribution is

(17) $$P\left(n\right)\propto n^{-\nu }e^{-n/a-b/n},$$

combining a power‐law section with exponent

(18) $$\nu =1-\frac{2r_{\mathrm{eff}}^{*}}{\gamma_{\mathrm{eff}}}$$

and downward ‘bends’ beyond sufficiently high or low abundances

(19) $$a=\frac{\gamma_{\mathrm{eff}}}{2\varepsilon },\;b=\frac{2\lambda }{\gamma_{\mathrm{eff}}}.$$

In the general case with finite noise correlation the distribution can be solved for approximately and is also a ‘bent’ power law (Appendix G).

If we consider ν (real), $\left(>0\right)$, and b $\left(>0\right)$ as independent parameters, then Equation (17) is known as the generalised inverse Gaussian (GIG) distribution (Jørgensen 1982). It contains as special or limiting cases: the inverse Gaussian distribution, the gamma distribution, a continuous interpolation of the powerbend distribution, pure power law, and the lognormal distribution. All of these (including the GIG itself (Sichel 1997)) have been considered as ‘underlying’ SADs in the ecological literature, commonly mixed with a Poisson distribution to model sampling effort (McGill et al. 2007). The principal differences between many alternative SADs is the presence and location of a mode (which may or may not be detectable in small‐size samples) and the extent and slope of a power law section.

For us, the GIG parameters ν,a,b are *not* independent, however. They depend on the effective parameters $r_{\mathrm{eff}}$ and $\gamma_{\mathrm{eff}}$, which are in turn determined implicitly through the complex community dynamics by the seven base parameters $S,r^{*},K\left(=r^{*}/\mu \right),\gamma ,\tau ,\varepsilon ,\lambda$. Of the possible shapes that the GIG affords, which are actually realised by the model, and how can we interpret the shape in terms of the underlying ecological processes? One approach to infer the effective parameters from the base set is by imposing a self‐consistency relation on the focal‐species model (see Appendix G for an explanation and tractable special case). In the following, we instead note that a dimensional analysis of Equation (1) (Appendix E) indicates that the variation in distribution shape is mainly captured by two composite parameters:

(20) $$B=\frac{\mathrm{S\lambda}}{\mathrm{K\gamma}}\;\text{and}\;\Sigma =\frac{\mathrm{K\varepsilon}}{\gamma }.$$

We interpret the first (B) as a *Buffering* dimension: it increases with the net immigration rate (Sλ). The second (Σ) we interpret as a *Stabilisation* dimension: it increases with the excess self‐regulation (ε). Both are reduced by increasing environmental noise (γ), which reflects the notion that the amount of buffering or stabilisation is relative to the strength of exclusionary processes. Increasing carrying capacity (K) lowers buffering, as the relative abundance increase from an immigrating individual is proportionally less, and increases stabilisation, as more species attain abundances where self‐regulation is strong.

We simulate 10′000 communities with base parameters drawn randomly from a wide range of values, and quantify the distribution shape by three indices (illustrated in Figure 5A): the width W in number of abundance decades; the modal abundance $n^{*}$; and the power‐law exponent ν as defined by Equation (18). By projecting these indeces in the Buffering–Stabilisation space, we confirm that the compound parameters B and Σ capture most of the variation in SAD shape Figure 5C. The shape varies continuously, but four main types can be identified (Figure 5B), corresponding to different ecological regimes.

#### 3.5.1 Exclusion regime

When buffering and stabilisation are both small, we observe a power‐law section spanning many orders of magnitude—a few species are highly dominant and the rest are rare. In the complete absence of buffering or stabilisation (as in Equation (5)), fluctuations drive an ever‐widening power law of exponent approaching 1 (Figure S3; see also the Poisson‐process limit law (Eliazar 2020)).

#### 3.5.2 Buffered Regime

Buffering increases the lower bound of abundances, and the power‐law section steepens as a larger fraction of species accumulate at the immigration threshold. Here we can find exponents ν in the upper empirical range.

#### 3.5.3 Stabilised Regime

With sufficient stabilisation, the mode of the distribution drastically shifts from lying close to the immigration threshold to approaching the single‐species carrying capacity (which is simultaneously reduced by self‐regulation). This shift coincides with the power‐law exponent changing sign. When it is positive, we observe a gamma distribution.

#### 3.5.4 Buffered‐and‐Stabilised Regime

When buffering and stabilisation are both prominent, abundances are tightly constrained between the lower limit due to immigration and an upper limit set by self‐regulation. The resulting shape is well approximated by a lognormal.

Because the SAD here reflects an FAD common to all species, its shape is closely linked to temporal beta diversity. The average Bray–Curtis similarity $\mathrm{BC}\left(t\right)$ of communities observed a time t apart (a quantity that has been applied to ecological time series (Fuhrman et al. 2015)), decays from 1 towards a limiting value that depends on the shape of the distribution, thus chiefly on the position in the Buffering–Stabilisation plane (Figure S4). Indeed, comparing the community composition at two time points sufficiently far apart amounts to randomising the species ranks of the second sample with respect to the first, while preserving the SAD. For the power‐law shape in the exclusion‐dominated region, the asymptotic similarity is smaller than when abundances are more narrowly distributed around their mode (<0.15 compared to >0.5). The *rate* at which BC similarity decays, however, depends on the actual value of the exclusion rate γ. For large values, specifically, the dominance of exclusion is associated to a faster species turnover and larger abundance fluctuations.

### 3.6 Heterogeneous Fitnesses Create Persistent Biases in Species Rarity

Finally, we relax the assumption of time‐averaged neutrality in order to test the robustness of our results when species within the same community differ in their long‐term abundance statistics, as expected in real communities.

**FIGURE 6:**
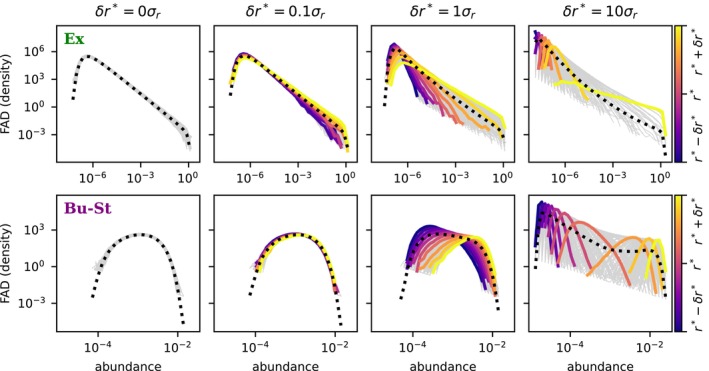
Emergence of frequent and infrequent species under breaking of time‐averaged neutrality. The two rows report the same numerical protocol but starting from different sets of model parameters, corresponding to the Exclusion regime and Buffered‐and‐Stabilised regime (points Ex and Bu‐St in Figure 5). Time‐averaged neutrality is broken by drawing the $r_{i}^{*}$s uniformly at random from the interval $\left[r^{*}-\delta r^{*},r^{*}+\delta r^{*}\right]$, with $\delta r^{*}$ varying by column. In each panel, the frequency–abundance distribution (FAD) of each individual species is plotted, with a subset of species shown in colour according to their $r_{i}^{*}$; the dotted dashed lines are the species‐averaged FADs (equal to the time‐averaged SAD). Simulation parameters used are S=500, $r^{*}=1$, K=1, γ=0.05, τ=10 and for Ex, Bu‐St, respectively, ε=0.05,50; $\lambda =10^{-8},3.2\times 10^{-5}$. Simulations were run for 500′000 time units.

We take two reference parameter sets corresponding to the Exclusion and Buffered‐and‐Stabilised regimes (same as in Figure 5). For each case, we draw the species‐specific fitness averages $r_{i}^{*}$ from a uniform distribution in the range $r^{*}\pm \delta r^{*}$, but keep all other parameters identical for all species. The fitness distributions are now same‐width normal distributions with different means; how much they overlap is controlled by $\delta r^{*}/\sigma_{r}$, which we vary from 0 (total overlap) to 10 (small overlap). As this ratio increases, the species‐specific FADs separate, and species of lower (higher) $r_{i}^{*}$ become biased towards rarity (dominance); see Figure 6. For the heavy‐tailed distribution in the Exclusion regime, the time‐averaged SAD (coinciding by definition with the species‐averaged FAD) changes little during this splitting. In the Buffered‐and‐stabilised regime, the initially lognormal time‐average SAD widens to develop a power‐law trend. The breaking of TAN also leads to a smaller turnover as measured by the BC index limit, as species become more constrained in their fluctuations, whether biased towards rarity or dominance (Figure S5).

Focussing on the emerging differences, we note that a spread in mean fitness within the limits of a doubling/halving of the average (the rightmost panels of Figure 6 have $r_{i}^{*}\in \left[0.5,1.5\right]$) is able to produce a distribution of species mean abundances spanning several orders of magnitude (Figure S6). The shape of the FADs can also differ between species. As FADs split, the ‘frequent’ species' FADs are more gamma‐like, whereas ‘infrequent’ ones have a longer right tail, as particularly evident in the St‐Bu example. The possible FAD shapes of different species are still described by the focal‐species model Equation (16), but now the species have different $r_{\mathrm{eff}}^{*}$, set by $r_{i}^{*}-\mu \bar{N}$. Thus, based on its expected fitness, a species may be relatively more or less constrained by immigration or self‐regulation.

## 4 Discussion

We have sought to understand how environmental stochasticity, intra‐ and interspecific competition and dispersal relate to three features of natural communities: the coexistence of many species, the commonness of rarity (reflected in heavy‐tailed SADs) and species turnover.

Even under the equalising assumptions of uniform competition and time‐averaged neutrality, environmental stochasticity drives the community towards ever‐greater unevenness (and species extinctions), unless countered by other processes. Contrary to strictly neutral dynamics driven by demographic noise (Kessler et al. 2015), the timescale on which a few species rise to dominance is independent of total carrying capacity (Dean and Shnerb 2020); its determinants are the variance of fitness fluctuations and their correlation time, beside a weak dependence on species richness. The stochastic exclusion dynamics reflect extreme‐value statistics that also underlie phase transitions in disordered systems in physics. The model thus reinforces the well‐subscribed notion that particular coexistence mechanisms are needed to explain natural diversity, at least in communities where competition is pervasive. We observed how increasing intraspecific suppression, or dispersal within a metacommunity (promoting a spatiotemporal buffering effect (Garcia Lorenzana et al. 2024; Loreau et al. 2003)) allows both absolute and effective species richness to be preserved on super‐generational timescales.

We then formulated a simple focal‐species model as a means to connect the fluctuation statistics of individual species with community statistics, especially the SAD. Under time‐average neutrality, it is a ‘bent power law’, close to the three‐parameter generalised inverse Gaussian distribution (GIG) postulated as a flexible SAD model over three decades ago (Sichel 1997). Emerging from our dynamical model, its parameters are not independent: instead we find that two compound parameters largely account for the variability in distribution shape. We interpret them as measures of *Buffering* (proportional to immigration rate) and *Stabilisation* (proportional to excess self‐regulation)—similar in spirit to dispersal‐limitation and niche–neutral axes (Gravel et al. 2006; Fisher and Mehta 2014; Haegeman and Loreau 2011; Leibovich et al. 2022), but measured with respect to strength of environmental fluctuations as opposed to neutral drift. The SAD approaches a wide power law of exponent ν=1 when both buffering and stabilisation are weak; stabilisation promotes a gamma distribution shape (ν<1, typically negative and producing a non‐zero mode); buffering makes the power law section narrower and steeper (ν>1); and the combination of both produces a peaked distribution well‐fitted by a lognormal. The focal‐species model lends an ecological interpretation to the exponent. It will be close to unity when mean effective fitness (encompassing its intrinsic growth rate and community interactions) is close to zero, or if effective environmental stochasticity is very large. Exponents ν>1, as for plankton, require *negative* mean effective fitness. In that case, species richness can only be sustained by immigration, which is indeed a major process of planktonic communities.

In reality, some species are persistently more common than others (Sæther et al. 2013). For example, an estuarine fish community contained both established ‘core’ species, and ‘occasional’, non‐establishing immigrants (Magurran and Henderson 2003). When we relaxed the assumption of time‐averaged neutrality by allowing some dispersion in species' expected fitness, we could produce order of magnitude differences in species mean abundance, and qualitatively different shapes of their frequency–abundance distributions (FADs), with implications for the SAD that remain to be systematically explored. The amplification of moderate difference in species demographic parameters into stark differences in their abundance patterns due to within‐community feedbacks speaks for the difficulty in predicting species‐level composition of natural communities. Other relevant sources of heterogeneity, for example, differences in intraspecific (Yenni et al. 2017; Rovere and Fox 2019) or interspecific interactions (Mallmin et al. 2024; Kessler and Shnerb 2025; May 1972; Buche et al. 2022), further add to this complexity.

As a highly aggregated measure, the SAD may provide limited information for disambiguating between alternative theories (McGill et al. 2007). A per‐species spectrum of FADs provides a richer picture (Grilli 2020), but requires highly resolved time‐series and does not give direct information about community timescales. Complementing the abundance distributions, we studied the decay in community similarity over time as an empirically relevant measure of turnover (Fuhrman et al. 2015; Kampichler and van der Jeugd 2012). In steady state, the long‐time similarity to an initial composition is higher in communities with a narrower SAD and where species are more constrained in their range of fluctuations due to long‐term differences in fitness. The decay rate is closely connected to the strength of environmental fluctuations. Investigating empirically if there are correlations between SAD shape parameters and turnover measures would be a worthwhile extension of recent macroecological surveys (Gao et al. 2025; Blowes et al. 2019; Pinsky et al. 2025).

Ultimately, comparing empirical community data and theoretical predictions over a whole suite of simultaneous patterns (abundance distributions, turnover, extinction times, species correlations, …) will elucidate community assembly. Such an endeavour is well underway for microbial communities (Grilli 2020; Descheemaeker et al. 2021; Ji et al. 2020; Descheemaeker and de Buyl 2020; Sireci et al. 2023; Wang and Liu 2023; Maskawa et al. 2025). Models have varied in the emphasis on species interactions (Wang and Liu 2023), parameter heterogeneity (Grilli 2020), or spatiotemporal coarse‐graining (Maskawa et al. 2025), but generally encompass fluctuating growth rates. Despite the differences in ecological mechanism, stochastic variation in intrinsic fitness, in interaction rates, or interaction‐driven chaos, leads to more or less identical forms of the focal species dynamics (Suweis et al. 2024; Mallmin et al. 2024; Arnoulx de Pirey and Bunin 2024). This ‘multiple realizability’ of fluctuating growth rates points towards the generality of our results.

## Author Contributions

Emil Mallmin conceived the study and carried out the formal analysis. All authors contributed to conceptual development and to writing the manuscript.

## Supporting information


**Data S1:** ele70333‐sup‐0001‐DataS1.pdf.

## Data Availability

Code used for simulation and plotting is publicly available at Zenodo: https://doi.org/10.5281/zenodo.15681474.

## Appendix A

### Statistics of Fitness Fluctuations

Integrating the Ornstein‐Uhlenbeck process, Equation (3), from time $t_{k}$ to $t_{k+1}=t_{k}+\Delta t$ (dropping the index i for brevity),

(21)
$$r\left(t_{k+1}\right)=r\left(t_{k}\right)e^{-\Delta t/\tau }+r^{*}\left(1-e^{-\Delta t/\tau }\right)+\sigma_{r}u\left(\Delta t/\tau \right)X_{k},$$

where $X_{k}∼N\left(0,1\right)$ and $u\left(s\right)=\sqrt{1-e^{-2s}}$. The integrated fitness satisfies (Gillespie 1996).

(22)
$$R\left(t_{k+1}\right)=R\left(t_{k}\right)+r^{*}\Delta t+\tau \left(r\left(t_{k}\right)-r^{*}\right)\left(1-e^{-\Delta t/\tau }\right)+\sqrt{\gamma \Delta t}v\left(\Delta t/\tau \right)Y_{k},$$

where $Y_{k}∼N\left(0,1\right)$ and

(23)
$$v\left(s\right)=\sqrt{1-\frac{1}{2s}\left(3+e^{-2s}-4e^{-s}\right)}∼\left\{\begin{array}{ll} \frac{1}{\sqrt{3}}s & s\ll 1\\ 1 & s\gg 1 \end{array}\right..$$

Note that $X_{k},Y_{k}$ have a correlation $C\left(\Delta t/\tau \right)$ with

(24)
$$C\left(s\right)=\frac{1-2e^{-s}+e^{-2s}}{\sqrt{\left(1-e^{-2s}\right)\left(2s-\left(3+e^{-2s}-4e^{-s}\right)\right)}},$$

which is also the correlation between $R\left(\Delta t\right)$ and $r\left(\Delta t\right)$. It takes t≈50τ for $C\left(t/\tau \right)$ to reduce from $C\left(0\right)=\sqrt{3}/2$ to a 10% correlation, and 5000τ to a 1% correlation.

## Appendix B

### Numerical Integration

The numerical integration of Equation (1) was done with the scheme

(25)
$$n_{i}\left[t+\Delta t\right]\leftarrow n_{i}\left[t\right]\exp\left\{\Delta R_{i}\left[t\right]-\Delta t\left(\mathrm{\mu N}\left[t\right]+\varepsilon n_{i}\left[t\right]\right)\right\}+\lambda \Delta t.$$

It ensures positivity of abundances, and, if ε=0 and λ=0, reproduces the analytically exact path probabilities for relative abundances regardless of step size Δt. A step‐size of Δt=0.01 was used throughout. For Ornstein‐Uhlenbeck noise, $\Delta R_{i}$ can be sampled from Equation (22) in tandem with Equation (21), involving no approximations.

In the metacommunity version of the model, Equation (15),

(26)
$$n_{\mathrm{i\alpha}}\left[t+\Delta t\right]\leftarrow \left(\sum_{\beta =1}^{M}n_{\mathrm{i\beta}}\left[t\right]D_{\beta \alpha }\right)\times \exp\left\{\Delta R_{\mathrm{i\alpha}}\left[t\right]-\Delta t\left(\mu N_{\alpha }\left[t\right]+\varepsilon n_{\mathrm{i\alpha}}\left[t\right]\right)\right\},$$

where

(27)
$$D_{\alpha \alpha }=1-e^{-d_{\alpha }\Delta t},\;D_{\alpha \beta }=\frac{d_{\beta \alpha }}{d_{\alpha }}e^{-d_{\alpha }\Delta t}\;\left(\beta \neq \alpha \right).$$

## Appendix C

### Derivation of the Replicator Equation

To derive Equations (7) and (8) from Equation (1), consider more generally:

(28)
$${\dot{n}}_{i}\left(t\right)=n_{i}\left(t\right)\left[r_{i}\left(t\right)-g\left(\vec{n}\left(t\right),\vec{r}\left(t\right),t\right)\right].$$

By summing over i we get immediately

(29)
$$\dot{N}\left(t\right)=N\left(t\right)\left[\rho \left(t\right)-g\left(\vec{n}\left(t\right),\vec{r}\left(t\right),t\right)\right],$$

with $\rho =\sum_{j}r_{j}n_{j}/N$. Applying the chain rule to $p_{i}=n_{i}/N$,

(30)
$${\dot{p}}_{i}=\frac{{\dot{n}}_{i}}{N}-p_{i}\frac{\dot{N}}{N}=\frac{n_{i}}{N}\left(r_{i}-g\right)-p_{i}\left(\rho -g\right)=p_{i}\left(r_{i}-\rho \right).$$

The cancellation of g hinges upon it being identical for all species (i.e., neutral).

## Appendix D

### Derivation of Focal‐Species Replicator Equation

Here we derive the focal‐species Equation (12) revealing the stickiness effect. With $p_{i}$, i=1,…,S, the relative abundances in the full community, define the relative abundance $p_{i\backslash 1}$, i=2,…,S, in the sub‐community excluding species 1:

(31)
$$p_{i\backslash 1}≔\frac{n_{i}}{\sum_{j=2}^{S}n_{j}}=\frac{p_{i}}{1-p_{1}},\;i>1.$$

Note that $p_{i\backslash 1}$ is independent of the fitness of species 1 since

(32)
$$p_{i\backslash 1}=\frac{p_{i}\left(0\right)e^{R_{i}}}{\sum_{j>1}p_{j}\left(0\right)e^{R_{j}}}.$$

The mean fitness in the full community is

(33)
$$\rho =p_{1}r_{1}+\sum_{i=2}^{S}p_{i}r_{i}=p_{1}r_{1}+\left(1-p_{1}\right)\rho_{\backslash 1},$$

where we have substituted $p_{i\backslash 1}\left(1-p_{1}\right)$ for $p_{i}$ according to Equation (31), and identified

(34)
$$\rho_{\backslash 1}≔\sum_{i=2}^{S}p_{i\backslash 1}r_{i}.$$

Substituting the above expression for ρ in the replicator equation, Equation (7), for i=1 reproduces Equation (12).

## Appendix E

### Unevenness as a Phase Transition

Here we give more details on the derivation of the timescale of the unevenness transition and the mapping to a spin glass.

At any fixed time t, the distribution of $p_{i}\left(t\right)$, Equation (10), has the mathematical form of a Boltzmann distribution,

(35)
$$p_{i}^{B}=\frac{e^{-\beta E_{i}}}{Z^{B}}.$$

The S species correspond to as many configurations of some physical system, for instance the $S=2^{J}$ possible configuration of J Ising spins, in contact with a heat bath at inverse temperature β. $\ln n_{i}\left(0\right)+R_{i}\left(t\right)$ is mapped to $\beta E_{i}$, with $E_{i}$ the energy of the physical configuration. We assume fitness fluctuations according to Equation (3); that $n_{i}\left(0\right)$ are log‐normally distributed with log‐variance $\sigma_{n,0}^{2}$; and that the $r_{i}\left(0\right)$s are normal with variance $\sigma_{r,0}^{2}$. Then $\ln n_{i}\left(0\right)+R_{i}\left(t\right)$ is normally distributed at any t≥0. The assumption of normal energy levels with variance J/2 defines the random energy model (REM) of a spin glass (Derrida 1980, 1981). The mapping from the ecological model to the REM is fixed by choosing $\beta =\beta \left(t\right)$ according to

(36)
$$\beta^{2}J/2=\mathrm{Var}\left[n\left(0\right)+R\left(t\right)\right],\;2^{J}=S.$$

In the ‘thermodynamic limit’ J→∞, the REM exhibits a condensation phase transition at the critical inverse temperature $\beta_{c}=2\sqrt{\ln2}$, separating a phase dominated by the energy ground state and one where almost all states are equiprobable. Thus, in the community model there is a dominance transition at the critical time $t_{c}$ given implicitly by $\beta_{c}=\beta \left(t_{c}\right)$. From our assumptions, using Equation (22),

(37)
$${\left(\frac{\beta \left(t\right)}{\beta_{c}}\right)}^{2}=\frac{1}{2\ln S}\left(\sigma_{n,0}^{2}+\tau^{2}{\left(1-e^{-t/\tau }\right)}^{2}\sigma_{r,0}^{2}+\gamma v^{2}\left(t/\tau \right)\right).$$

In the case of an initially even community, $\sigma_{n,0}=0$, and initial fitnesses drawn from the stationary distribution, $\sigma_{r,0}=\sigma_{r}$, we have

(38)
$$\gamma t_{c}w^{2}\left(t_{c}/\tau \right)=2\ln S,$$

where

(39)
$$w^{2}\left(s\right)≔\frac{{\left(1-e^{-s}\right)}^{2}}{s}+v^{2}\left(s\right)=\frac{e^{-2s}-\left(1-2s\right)}{2s}.$$

Using $w\left(s\gg 1\right)=1$ and $w\left(s\right)=s+O\left(s^{2}\right)$,

(40)
$$t_{c}=\left\{\begin{array}{ll} \frac{2\ln S}{\gamma }, & \tau \ll t_{c},\\ \frac{2\ln S}{\gamma }\cdot \sqrt{\frac{\gamma \tau }{2\ln S}}, & \tau \gg t_{c}. \end{array}\right.$$

Note, however, that the critical time should diverge in the large‐community limit where the condensation phase transition becomes sharp; for finite communities the unevenness transition is therefore always observed as a smooth crossover of regimes.

For a heuristic derivation of Equation (38), following (Derrida 1981), consider, at any given time, the variance in the normalisation factor Z in Equation (10) over many realisations of the fluctuating fitnesses: if it is large, that signals that the sum tends to be dominated by a few fluctuating terms. Denote $X_{i}\left(t\right)=\ln n_{i}\left(0\right)+R_{i}\left(t\right)$. The analysis is straightforward if the $X_{i}\left(t\right)$ are normal i.i.d. Then using $E\left[e^{X_{i}}\right]=\exp\left\{E\left[X_{i}\right]+\mathrm{Var}\left[X\right]/2\right\}$,

(41)
$$E\left[Z\right]=Se^{E\left[X\right]+\frac{1}{2}\mathrm{Var}\left[X\right]},$$

(42)
$$E\left[Z^{2}\right]=Se^{2E\left[X\right]}\left(e^{2\mathrm{Var}\left[X\right]}+\left(S-1\right)e^{\mathrm{Var}\left[X\right]}\right);$$

hence, with S−1≈S,

(43)
$$\frac{\mathrm{Var}\left[Z\right]}{E{\left[Z\right]}^{2}}=e^{\mathrm{Var}\left[X\right]-\ln S}.$$

As S becomes large, the Z‐variance is non‐negligible for times t large enough that the above exponent is not very negative; $\mathrm{Var}\left[X\left(t\right)\right]≳\ln S$.

## Appendix F

### Dimensional Analysis

Here we show which parameter combinations have qualitative effects on the model Equations (1) and (3).

The special case Equation (5) indicates that 1/γ (rather than $1/r^{*}$) is generally the natural timescale, and $K=r^{*}/\mu$ the natural scale of abundances. We therefore introduce the non‐dimensional time $\tilde{t}≔\mathrm{\gamma t}$. We shift and rescale fitnesses as ${\tilde{r}}_{i}\left(\tilde{t}\right)≔\left(r_{i}\left(t\right)-r^{*}\right)/\gamma$, and define $\tilde{W}\left(\tilde{t}\right)∼N\left(0,\tilde{t}\right)$, to transform Equation (3) into

(44)
$$\left(\tau \gamma \right)\frac{d}{d\tilde{t}}{\tilde{r}}_{i}=-{\tilde{r}}_{i}+\frac{d}{d\tilde{t}}\tilde{W}.$$

We derive evolutions for $\tilde{N}\left(\tilde{t}\right)≔N\left(t\right)/K$ and ${\tilde{p}}_{i}\left(\tilde{t}\right)≔p_{i}\left(t\right)$, similar to the derivation of Equations (8 and 7) in Appendix C, obtaining

(45)
$$\frac{d}{d\tilde{t}}\tilde{N}=\frac{r^{*}}{\gamma }\tilde{N}\left[1-\left(1+\frac{\mathrm{K\varepsilon}}{r^{*}}\frac{1}{{\tilde{S}}_{\mathrm{eff}}}\right)\tilde{N}+\tilde{\rho }\right]+\frac{\mathrm{S\lambda}}{\mathrm{\gamma K}},$$

and

(46)
$$\frac{d}{d\tilde{t}}{\tilde{p}}_{i}={\tilde{p}}_{i}\left[{\tilde{r}}_{i}-\tilde{\rho }\right]+\frac{\mathrm{K\varepsilon}}{\gamma }\tilde{N}{\tilde{p}}_{i}\left(\frac{1}{{\tilde{S}}_{\mathrm{eff}}}-{\tilde{p}}_{i}\right)+\frac{\mathrm{S\lambda}}{\mathrm{\gamma K}}\cdot \tilde{N}\left(\frac{1}{S}-{\tilde{p}}_{i}\right),$$

where $\tilde{\rho }≔\sum_{i}\tilde{r_{i}}{\tilde{p}}_{i}$. As is to be expected, the set of Equations ((44), (45), (46)–(44), (45), (46)) depend (beside S) on the full set of non‐dimensionalized parameters

(47)
$$\tilde{\tau }≔\gamma \tau ,\;{\tilde{r}}^{*}≔\frac{r^{*}}{\gamma },\;\tilde{\varepsilon }≔\frac{\mathrm{K\varepsilon}}{\gamma },\;{\tilde{\lambda }}_{\mathrm{tot}}≔\frac{\mathrm{S\lambda}}{\mathrm{\gamma K}}.$$

However, Equation (46) only has a *direct* dependence on $\tilde{\varepsilon }$ and ${\tilde{\lambda }}_{\mathrm{tot}}$. So, if $\tilde{N}$ is relatively constant and τ not much larger than 1/γ we can expect the stationary distribution to depend mainly on $\tilde{\varepsilon }$ and ${\tilde{\lambda }}_{\mathrm{tot}}$. To highlight their importance and interpretation we denote them instead by Σ and B, Equation (20).

As a complementary understanding of the reduced parameter space, consider rescaling time by $r^{*}$ (instead of γ) and define

(48)
$$\hat{\gamma }≔\frac{\gamma }{r^{*}},\;\hat{\varepsilon }=\frac{\mathrm{K\varepsilon}}{r^{*}},\;{\hat{\lambda }}_{\mathrm{tot}}=\frac{\mathrm{S\lambda}}{Kr^{*}}.$$

Then $\Sigma =\hat{\varepsilon }/\hat{\gamma }$ and $B={\hat{\lambda }}_{\mathrm{tot}}/\hat{\gamma }$. The plane $\left(B,\Sigma \right)$ can then be mapped into the simplex defined by the relative proportions of the compound parameters Equation (48):

(49a)
$$f_{\gamma }≔\frac{\hat{\gamma }}{\hat{\gamma }+\hat{\varepsilon }+{\hat{\lambda }}_{\mathrm{tot}}}=\frac{1}{1+\Sigma +B},$$

(49b)
$$f_{\epsilon }≔\frac{\hat{\varepsilon }}{\hat{\gamma }+\hat{\varepsilon }+{\hat{\lambda }}_{\mathrm{tot}}}=\frac{\Sigma }{1+\Sigma +B},$$

(49c)
$$f_{\lambda }≔\frac{{\hat{\lambda }}_{\mathrm{tot}}}{\hat{\gamma }+\hat{\varepsilon }+{\hat{\lambda }}_{\mathrm{tot}}}=\frac{B}{1+\Sigma +B},$$

and, conversely, $\Sigma =f_{\varepsilon }/f_{\gamma }$, $B=f_{\lambda }/f_{\gamma }$. That is, what matters for the shape of static abundance patterns (e.g., SAD) is essentially the relative strength of exclusion rate, excess self‐limitation, and immigration.

## Appendix G Stationary Abundance Distribution of the Focal‐Species Model

Matching the statistics of $r_{\mathrm{eff}}\left(t\right)$ to $r_{i}\left(t\right)-\mathrm{\mu N}\left(t\right)$ (for some arbitrary i), we set its mean to

(50)
$$r_{\mathrm{eff}}^{*}=r^{*}-\mu \bar{N},$$

with the over bar denoting an average over long times; the variance (neglecting the small covariance of focal species fitness and total abundance) to

(51)
$$\sigma_{r_{\mathrm{eff}}}^{2}\approx \sigma_{r}^{2}+\mu^{2}\mathrm{Var}\left[N\right];$$

and $\tau_{\mathrm{eff}}\approx \tau$, because fluctuations are chiefly driven by $r_{i}\left(t\right)$. Taking the noise statistics as given, the focal‐species model Equation (16) is a one‐dimensional SDE whose stationary distribution can be obtained by standard techniques. When the noise is coloured, an approximate solution (see SI of (Mallmin et al. 2024) for derivation) is given by

(52)
$$\begin{array}{c} P\left(n\right)\approx \frac{1}{N}\left(\frac{1}{\tau_{\mathrm{eff}}}+\mathrm{\varepsilon n}+\frac{\lambda }{n}\right)n^{-\nu }\;\exp\left[-q_{+}\left(\mathrm{\varepsilon n}\right)-q_{-}\left(\lambda /n\right)\right]\\ \nu =1-\frac{2r_{\mathrm{eff}}^{*}}{\gamma_{\mathrm{eff}}},\;q_{\pm }\left(x\right)=\frac{\tau_{\mathrm{eff}}}{\gamma_{\mathrm{eff}}}{\left(x+\tau_{\mathrm{eff}}^{-1}\pm r_{\mathrm{eff}}^{*}\right)}^{2} \end{array}.$$

In the fast‐environment limit (τ→0 at fixed γ), we obtain Equation (17) (which is the exact solution) by keeping from the large parenthesis in Equation (52) only the constant diverging term, and expanding $q_{\pm }\left(x\right)$ to first finite order; apparent divergences of constants must cancel in the new normalisation.

The normalisation factor corresponding to Equation (17) is that of the generalised inverse Gaussian (Jørgensen 1982),

(53)
$$N_{\text{fast}-\mathrm{env}}=2{\left(\mathrm{ab}\right)}^{-\frac{1-\nu }{2}}K_{1-\nu }\left(2\sqrt{b/a}\right),$$

where $K_{p}\left(z\right)$ is the p‐th order modified Bessel function of the second kind. The normalisation factor N for Equation (52) must be evaluated by numerical integration, and requires special care. Write Equation (52) as

(54)
$$P\left(n\right)=e^{H\left(n\right)-\ln N},$$

and evaluate lnN according to

(55)
$$\ln N=L^{*}+\ln\int_{-\infty }^{\infty }dy\;e^{L\left(y\right)-L^{*}}$$

where $L\left(y\right)=H\left(e^{y}\right)+y$ and $L^{*}=\max_{y}L\left(y\right)$. The integral appearing in Equation (55) can be evaluated with standard numerical integration.

In principle, the values of the effective parameters $r_{\mathrm{eff}}^{*}$, $\gamma_{\mathrm{eff}}$, $\tau_{\mathrm{eff}}$ could be obtained through solving self‐consistency relations, rather than being extracted from simulation, at least for such values of the original parameters that fluctuations in $N\left(t\right)$ are small. To illustrate, consider the special case of no immigration and the fast‐environment limit, and suppose $N\left(t\right)\approx \bar{N}$. The focal species then undergoes stochastic logistic growth and $P\left(n\right)$ is the PDF of the Gamma distribution with shape parameter $2r_{\mathrm{eff}}^{*}/\gamma$ and scale parameter γ/2ε. On the one hand, since species fluctuate effectively independently, we posit

(56)
$$\bar{N}=S\int_{0}^{\infty }\mathrm{nP}\left(n\right)dn,$$

which evaluates to $Sr_{\mathrm{eff}}^{*}/\varepsilon$; on the other hand, we have the definition of $r_{\mathrm{eff}}^{*}$ via $\bar{N}$, Equation (50). Combining these relations we find

(57)
$$r_{\mathrm{eff}}^{*}=r^{*}\left(\frac{1}{1+\frac{\mathrm{\mu S}}{\varepsilon }}\right).$$

The solution is valid (the Gamma distribution remains within its range of normalizability) as long as ε is positive. The solution is lost when ϵ→0, as we indeed expect from our analysis of Equation (5).

## Appendix H

### Coexistence Fixed Point for Fixed Fitness Values

We are solving for the fixed points of

(58)
$${\dot{n}}_{i}\left(t\right)=n_{i}\left(t\right)\left[r_{i}-\mathrm{\mu N}\left(t\right)-\varepsilon n_{i}\left(t\right)\right]=0,\;\varepsilon >0,$$

where $r_{i}$ are some fixed numbers. Let $s_{i}=1$ if species i survives in the fixed point and 0 if it is extinct. Then its equilibrium abundance is

(59)
$${\hat{n}}_{i}=s_{i}\frac{r_{i}-\mu \hat{N}}{\varepsilon },$$

where $\hat{N}=\sum_{j=1}^{S}{\hat{n}}_{j}$. Summing Equation (59) and solving for $\hat{N}$ yields

(60)
$$\hat{N}=\frac{\hat{r}}{\mu +\varepsilon /\hat{S}},$$

where $\hat{S}=\sum_{j=1}^{S}s_{j}$ and $\hat{r}=\sum_{j=1}^{S}s_{j}r_{j}/\hat{S}$. Thus

(61)
$${\hat{n}}_{i}=s_{i}\frac{1}{\varepsilon }\left(r_{i}-\frac{\hat{r}}{1+\frac{\varepsilon }{\mu \hat{S}}}\right).$$

Feasibility requires that $r_{i}>\hat{r}/\left(1+\varepsilon /\mu \hat{S}\right)$ if $s_{i}=1$; uninvadability requires that $s_{i}=1$ if this rate inequality holds. Stability of fixed points follows from the negative definiteness of the interaction matrix $\left[-\mu -\delta_{\mathrm{ij}}\varepsilon \right]$; symmetric interactions preclude chaos or limit cycles (Hofbauer and Sigmund 2002). Suppose $r_{1}>r_{2}>r_{3}\ldots$. One concludes that there is a unique uninvadable fixed point consisting of the species with the largest $r_{i}$, up to the largest index i for which $r_{i}>\sum_{j=1}^{i}r_{j}/\left(1+\varepsilon /\mathrm{\mu i}\right)$.

If the $r_{i}$ were drawn from $N\left(r^{*},\sigma_{r}\right)$, then the smallest among S would be $r_{S}=r-h\left(S\right)\sigma_{r}$, where $h\left(S\right)$ is a random variable whose distribution for large S is known from extreme value theory (Vivo 2015). For S=100,1000,10000, $E\left[h\left(S\right)\right]\approx 2.5,3.2,3.9$, that is, relatively insensitive to S. Thus, essentially all species coexist when

(62)
$$\frac{\varepsilon }{\mu }>S\left(\frac{1}{1-h\left(S\right)\sigma_{r}/\hat{r}}-1\right)\approx 3S\frac{\sigma_{r}}{r^{*}},$$

if $\sigma_{r}/r^{*}$ is small.

## References

[ele70333-bib-0001] Advani, M. , G. Bunin , and P. Mehta . 2018. “Statistical Physics of Community Ecology: A Cavity Solution to Macarthur's Consumer Resource Model.” Journal of Statistical Mechanics: Theory and Experiment 2018: 033406. 30636966 10.1088/1742-5468/aab04e PMC6329381

[ele70333-bib-0015] Arnoulx de Pirey, T. , and G. Bunin . 2023. “Aging by Near‐Extinctions in Many‐Variable Interacting Populations.” Physical Review Letters 130: 098401. 36930904 10.1103/PhysRevLett.130.098401

[ele70333-bib-0016] Arnoulx de Pirey, T. , and G. Bunin . 2024. “Many‐Species Ecological Fluctuations as a Jump Process From the Brink of Extinction.” Physical Review X 14: 011037.

[ele70333-bib-0002] Barabás, G. , R. D'Andrea , and S. M. Stump . 2018. “Chesson's Coexistence Theory.” Ecological Monographs 88: 277.

[ele70333-bib-0003] Barbier, M. , and J.‐F. Arnoldi . 2017. “The Cavity Method for Community Ecology.” Preprint, bioRxiv.

[ele70333-bib-0004] Barbier, M. , J.‐F. Arnoldi , G. Bunin , and M. Loreau . 2018. “Generic Assembly Patterns in Complex Ecological Communities.” PNAS 115: 2156. 29440487 10.1073/pnas.1710352115 PMC5834670

[ele70333-bib-0005] Blowes, S. A. , S. R. Supp , L. H. Antão , et al. 2019. “The Geography of Biodiversity Change in Marine and Terrestrial Assemblages.” Science 366: 339. 31624208 10.1126/science.aaw1620

[ele70333-bib-0006] Blumenthal, E. , J. W. Rocks , and P. Mehta . 2024. “Phase Transition to Chaos in Complex Ecosystems With Nonreciprocal Species‐Resource Interactions.” Physical Review Letters 132: 127401. 38579223 10.1103/PhysRevLett.132.127401 PMC11780484

[ele70333-bib-0007] Buche, L. , J. W. Spaak , J. Jarillo , and F. De Laender . 2022. “Niche Differences, Not Fitness Differences, Explain Predicted Coexistence Across Ecological Groups.” Journal of Ecology 110: 2785.

[ele70333-bib-0008] Bunin, G. 2017. “Ecological Communities With Lotka‐Volterra Dynamics.” Physical Review E 95: 042414. 28505745 10.1103/PhysRevE.95.042414

[ele70333-bib-0009] Chesson, P. 2000. “Mechanisms of Maintenance of Species Diversity.” Annual Review of Ecology and Systematics 31: 343.

[ele70333-bib-0010] Chesson, P. , and J. J. Kuang . 2008. “The Interaction Between Predation and Competition.” Nature 456: 235. 19005554 10.1038/nature07248

[ele70333-bib-0011] Chesson, P. L. , and R. R. Warner . 1981. “Environmental Variability Promotes Coexistence in Lottery Competitive Systems.” American Naturalist 117: 923.

[ele70333-bib-0012] Cui, W. , R. Marsland , and P. Mehta . 2021. “Diverse Communities Behave Like Typical Random Ecosystems.” Physical Review E 104: 034416. 34654170 10.1103/PhysRevE.104.034416 PMC9005152

[ele70333-bib-0013] Dalmedigos, I. , and G. Bunin . 2020. “Dynamical Persistence in High‐Diversity Resource‐Consumer Communities.” PLoS Computational Biology 16: e1008189. 33044951 10.1371/journal.pcbi.1008189 PMC7581001

[ele70333-bib-0014] Danino, M. , and N. M. Shnerb . 2018. “Theory of Time‐Averaged Neutral Dynamics With Environmental Stochasticity.” Physical Review E 97: 042406. 29758719 10.1103/PhysRevE.97.042406

[ele70333-bib-0017] Dean, A. M. , and N. M. Shnerb . 2020. “Stochasticity‐Induced Stabilization in Ecology and Evolution: A New Synthesis.” Ecology 101: e03098. 32443176 10.1002/ecy.3098

[ele70333-bib-0018] Derrida, B. 1980. “Random‐Energy Model: Limit of a Family of Disordered Models.” Physical Review Letters 45: 79.

[ele70333-bib-0019] Derrida, B. 1981. “Random‐Energy Model: An Exactly Solvable Model of Disordered Systems.” Physical Review B 24: 2613.

[ele70333-bib-0020] Descheemaeker, L. , and S. de Buyl . 2020. “Stochastic Logistic Models Reproduce Experimental Time Series of Microbial Communities.” eLife 9: e55650. 32687052 10.7554/eLife.55650 PMC7410486

[ele70333-bib-0021] Descheemaeker, L. , J. Grilli , and S. de Buyl . 2021. “Heavy‐Tailed Abundance Distributions From Stochastic Lotka‐Volterra Models.” Physical Review E 104: 034404. 34654137 10.1103/PhysRevE.104.034404

[ele70333-bib-0022] Eliazar, I. 2020. Power Laws: A Statistical Trek. Springer International Publishing.

[ele70333-bib-0023] Engen, S. , and R. Lande . 1996. “Population Dynamic Models Generating Species Abundance Distributions of the Gamma Type.” Journal of Theoretical Biology 178: 325. 10.1016/0025-5564(95)00054-2 8714412

[ele70333-bib-0024] Enquist, B. J. , X. Feng , B. Boyle , et al. 2019. “The Commonness of Rarity: Global and Future Distribution of Rarity Across Land Plants.” Science Advances 5: eaaz0414. 31807712 10.1126/sciadv.aaz0414 PMC6881168

[ele70333-bib-0025] Fisher, C. K. , and P. Mehta . 2014. “The Transition Between the Niche and Neutral Regimes in Ecology.” Proceedings of the National Academy of Sciences 111: 13111. 10.1073/pnas.1405637111 PMC4246938 25157131

[ele70333-bib-0026] Fuhrman, J. A. , J. A. Cram , and D. M. Needham . 2015. “Marine Microbial Community Dynamics and Their Ecological Interpretation.” Nature Reviews Microbiology 13: 133. 25659323 10.1038/nrmicro3417

[ele70333-bib-0027] Gao, Y. , A. Abdullah , and M. Wu . 2025. “The Powerbend Distribution Provides a Unified Model for the Species Abundance Distribution Across Animals, Plants and Microbes.” Nature Communications 16: 4035. 10.1038/s41467-025-59253-9 PMC12041394 40301372

[ele70333-bib-0028] Garcia Lorenzana, G. , A. Altieri , and G. Biroli . 2024. “Interactions and Migration Rescuing Ecological Diversity.” PRX Life 2: 013014.

[ele70333-bib-0029] Gillespie, D. T. 1996. “Exact Numerical Simulation of the Ornstein‐Uhlenbeck Process and Its Integral.” Physical Review E 54: 2084. 10.1103/physreve.54.2084 9965289

[ele70333-bib-0030] Gravel, D. , C. D. Canham , M. Beaudet , and C. Messier . 2006. “Reconciling Niche and Neutrality: The Continuum Hypothesis.” Ecology Letters 9: 399. 16623725 10.1111/j.1461-0248.2006.00884.x

[ele70333-bib-0031] Grilli, J. 2020. “Macroecological Laws Describe Variation and Diversity in Microbial Communities.” Nature Communications 11: 4743. 10.1038/s41467-020-18529-y PMC7506021 32958773

[ele70333-bib-0032] Haegeman, B. , and M. Loreau . 2011. “A Mathematical Synthesis of Niche and Neutral Theories in Community Ecology.” Journal of Theoretical Biology 269: 150. 20946903 10.1016/j.jtbi.2010.10.006

[ele70333-bib-0033] Hardin, G. 1960. “The Competitive Exclusion Principle.” Science 131: 1292–1297. 14399717 10.1126/science.131.3409.1292

[ele70333-bib-0034] Hofbauer, J. , and K. Sigmund . 2002. Evolutionary Games and Population Dynamics. Cambridge University Press.

[ele70333-bib-0035] Hubbell, S. P. 2001. The Unified Neutral Theory of Biogeography. Princeton University Press.

[ele70333-bib-0036] Hubbell, S. P. 2005. “Neutral Theory in Community Ecology and the Hypothesis of Functional Equivalence.” Functional Ecology 19: 166.

[ele70333-bib-0037] Hutchinson, G. E. 1961. “The Paradox of the Plankton.” American Naturalist 95: 137–145.

[ele70333-bib-0038] Ji, B. W. , R. U. Sheth , P. D. Dixit , K. Tchourine , and D. Vitkup . 2020. “Macroecological Dynamics of Gut Microbiota.” Nature Microbiology 5: 768. 10.1038/s41564-020-0685-1 32284567

[ele70333-bib-0039] Johnson, E. C. , and A. Hastings . 2022. “Towards a Heuristic Understanding of the Storage Effect.” Ecology Letters 25: 2347. 36181717 10.1111/ele.14112

[ele70333-bib-0040] Jørgensen, B. 1982. Statistical Properties of the Generalized Inverse Gaussian Distribution. Springer.

[ele70333-bib-0041] Kalyuzhny, M. , R. Kadmon , and N. M. Shnerb . 2015. “A Neutral Theory With Environmental Stochasticity Explains Static and Dynamic Properties of Ecological Communities.” Ecology Letters 18: 572. 25903067 10.1111/ele.12439

[ele70333-bib-0042] Kampichler, C. , and H. P. van der Jeugd . 2012. “Determining Patterns of Variability in Ecological Communities: Time Lag Analysis Revisited.” Environmental and Ecological Statistics 20: 271.

[ele70333-bib-0043] Kessler, D. , S. Suweis , M. Formentin , and N. M. Shnerb . 2015. “Neutral Dynamics With Environmental Noise: Age‐Size Statistics and Species Lifetimes.” Physical Review E 92: 022722. 10.1103/PhysRevE.92.022722 26382447

[ele70333-bib-0044] Kessler, D. A. , and N. M. Shnerb . 2025. “The Dominant–Egalitarian Transition in Species‐Rich Communities.” eLife 14: e103999. 40694498 10.7554/eLife.103999 PMC12520658

[ele70333-bib-0045] Lande, R. , S. Engen , and B.‐E. Saether . 2003. Stochastic Population Dynamics in Ecology and Conservation. Oxford University Press.

[ele70333-bib-0046] Leibovich, N. , J. Rothschild , S. Goyal , and A. Zilman . 2022. “Phenomenology and Dynamics of Competitive Ecosystems Beyond the Niche‐Neutral Regimes.” Proceedings of the National Academy of Sciences 119, no. 43: e2204394119. 10.1073/pnas.2204394119 PMC9618050 36251996

[ele70333-bib-0047] Levin, S. A. 1970. “Community Equilibria and Stability, and an Extension of the Competitive Exclusion Principle.” American Naturalist 104: 413–423.

[ele70333-bib-0048] Loreau, M. , N. Mouquet , and A. Gonzalez . 2003. “Biodiversity as Spatial Insurance in Heterogeneous Landscapes.” Proceedings of the National Academy of Sciences 100: 12765. 10.1073/pnas.2235465100 PMC240692 14569008

[ele70333-bib-0049] MacArthur, R. H. , and E. O. Wilson . 1967. The Theory of Island Biogeography. Vol. 1. Princeton University Press.

[ele70333-bib-0050] Magurran, A. E. , and P. A. Henderson . 2003. “Explaining the Excess of Rare Species in Natural Species Abundance Distributions.” Nature 422: 714. 12700760 10.1038/nature01547

[ele70333-bib-0051] Malcai, O. , O. Biham , P. Richmond , and S. Solomon . 2002. “Theoretical Analysis and Simulations of the Generalized Lotka‐Volterra Model.” Physical Review E 66: 031102. 10.1103/PhysRevE.66.031102 12366094

[ele70333-bib-0052] Mallmin, E. , A. Traulsen , and S. De Monte . 2024. “Chaotic Turnover of Rare and Abundant Species in a Strongly Interacting Model Community.” Proceedings of the National Academy of Sciences 121: e2312822121. 10.1073/pnas.2312822121 PMC10945849 38437535

[ele70333-bib-0053] Maskawa, R. , H. Takayasu , L. Takayasu , W. Suda , and M. Takayasu . 2025. “Stochastic Spatiotemporal Growth Model Reproducing the Universal Statistical Laws of the Gut Microbiome.” Physical Review Research 7: 013269.

[ele70333-bib-0054] May, R. 1972. “Will a Large Complex System Be Stable?” Nature 238: 413. 4559589 10.1038/238413a0

[ele70333-bib-0055] McGill, B. 2003. “Strong and Weak Tests of Macroecological Theory.” Oikos 102: 679.

[ele70333-bib-0056] McGill, B. J. 2011. Biological Diversity: Frontiers in Measurement and Assessment, edited by A. E. Magurran and B. J. McGill . Oxford University Press.

[ele70333-bib-0057] McGill, B. J. 2018. “The What, How and Why of Doing Macroecology.” Global Ecology and Biogeography 28: 6.

[ele70333-bib-0058] McGill, B. J. , R. S. Etienne , J. S. Gray , et al. 2007. “Species Abundance Distributions: Moving Beyond Single Prediction Theories to Integration Within an Ecological Framework.” Ecology Letters 10: 995. 17845298 10.1111/j.1461-0248.2007.01094.x

[ele70333-bib-0059] Melbinger, A. , and M. Vergassola . 2015. “The Impact of Environmental Fluctuations on Evolutionary Fitness Functions.” Scientific Reports 5: 15211. 26477392 10.1038/srep15211 PMC4609966

[ele70333-bib-0060] Mutshinda, C. M. , Z. V. Finkel , C. E. Widdicombe , and A. J. Irwin . 2016. “Ecological Equivalence of Species Within Phytoplankton Functional Groups.” Functional Ecology 30: 1714.

[ele70333-bib-0061] Nee, S. 2005. “The Neutral Theory of Biodiversity: Do the Numbers Add Up?” Functional Ecology 19: 173.

[ele70333-bib-0062] Pande, J. , and N. M. Shnerb . 2022. “How Temporal Environmental Stochasticity Affects Species Richness: Destabilization, Neutralization and the Storage Effect.” Journal of Theoretical Biology 539: 111053. 35151719 10.1016/j.jtbi.2022.111053

[ele70333-bib-0063] Pearce, M. T. , A. Agarwala , and D. S. Fisher . 2020. “Stabilization of Extensive Fine‐Scale Diversity by Ecologically Driven Spatiotemporal Chaos.” PNAS 117: 14572–14583. 32518107 10.1073/pnas.1915313117 PMC7322069

[ele70333-bib-0064] Pesce, G. , A. McDaniel , S. Hottovy , J. Wehr , and G. Volpe . 2013. “Stratonovich‐To‐itô Transition in Noisy Systems With Multiplicative Feedback.” Nature Communications 4: 3733. 10.1038/ncomms3733 24217466

[ele70333-bib-0065] Pinsky, M. L. , H. Hillebrand , J. M. Chase , et al. 2025. “Warming and Cooling Catalyse Widespread Temporal Turnover in Biodiversity.” Nature 638: 995. 39880943 10.1038/s41586-024-08456-z

[ele70333-bib-0066] Preston, F. W. 1948. “The Commonness, and Rarity, of Species.” Ecology 29: 254–283.

[ele70333-bib-0067] Pueyo, S. 2006. “Diversity: Between Neutrality and Structure.” Oikos 112: 392.

[ele70333-bib-0068] Rogers, T. L. , S. B. Munch , S. i. S. Matsuzaki , and C. C. Symons . 2023. “Intermittent Instability Is Widespread in Plankton Communities.” Ecology Letters 26: 470. 36707927 10.1111/ele.14168

[ele70333-bib-0069] Rovere, J. , and J. W. Fox . 2019. “Persistently Rare Species Experience Stronger Negative Frequency Dependence Than Common Species: A Statistical Attractor That Is Hard to Avoid.” Global Ecology and Biogeography 28: 508.

[ele70333-bib-0070] Roy, F. , M. Barbier , G. Biroli , and G. Bunin . 2020. “Complex Interactions Can Create Persistent Fluctuations in High‐Diversity Ecosystems.” PLoS Computational Biology 16: e1007827. 32413026 10.1371/journal.pcbi.1007827 PMC7228057

[ele70333-bib-0071] Sæther, B. , S. Engen , and V. Grøtan . 2013. “Species Diversity and Community Similarity in Fluctuating Environments: Parametric Approaches Using Species Abundance Distributions.” Journal of Animal Ecology 82: 721. 23578202 10.1111/1365-2656.12068

[ele70333-bib-0072] Ser‐Giacomi, E. , L. Zinger , S. Malviya , et al. 2018. “Ubiquitous Abundance Distribution of Non‐Dominant Plankton Across the Global Ocean.” Nature Ecology & Evolution 2: 1243. 29915345 10.1038/s41559-018-0587-2

[ele70333-bib-0073] Sichel, H. S. 1997. “Modelling Species‐Abundance Frequencies and Species‐Individual Functions With the Generalized Inverse Gaussian‐Poisson Distribution.” South African Statistical Journal 31: 13–37.

[ele70333-bib-0074] Sireci, M. , M. A. Muñoz , and J. Grilli . 2023. “Environmental Fluctuations Explain the Universal Decay of Species‐Abundance Correlations With Phylogenetic Distance.” Proceedings of the National Academy of Sciences 120: e2217144120. 10.1073/pnas.2217144120 PMC10500273 37669363

[ele70333-bib-0075] Smetacek, V. 2012. “Making Sense of Ocean Biota: How Evolution and Biodiversity of Land Organisms Differ From That of the Plankton.” Journal of Biosciences 37: 589. 22922185 10.1007/s12038-012-9240-4

[ele70333-bib-0076] Soininen, J. 2014. “A Quantitative Analysis of Species Sorting Across Organisms and Ecosystems.” Ecology 95: 3284.

[ele70333-bib-0077] Suweis, S. , F. Ferraro , C. Grilletta , S. Azaele , and A. Maritan . 2024. “Generalized Lotka‐Volterra Systems With Time Correlated Stochastic Interactions.” Physical Review Letters 133: 167101. 39485958 10.1103/PhysRevLett.133.167101

[ele70333-bib-0078] Vallade, M. , and B. Houchmandzadeh . 2003. “Analytical Solution of a Neutral Model of Biodiversity.” Physical Review E 68: 061902. 10.1103/PhysRevE.68.061902 14754229

[ele70333-bib-0079] van Nes, E. H. , D. G. F. Pujoni , S. A. Shetty , G. Straatsma , W. M. de Vos , and M. Scheffer . 2024. “A Tiny Fraction of All Species Forms Most of Nature: Rarity as a Sticky State.” Proceedings of the National Academy of Sciences 121: e2221791120. 10.1073/pnas.2221791120 PMC10786311 38165929

[ele70333-bib-0080] Vellend, M. 2016. The Theory of Ecological Communities. Princeton University Press.

[ele70333-bib-0081] Vivo, P. 2015. “Large Deviations of the Maximum of Independent and Identically Distributed Random Variables.” European Journal of Physics 36: 055037.

[ele70333-bib-0082] Volkov, I. , J. R. Banavar , S. P. Hubbell , and A. Maritan . 2003. “Neutral Theory and Relative Species Abundance in Ecology.” Nature 424: 1035. 12944964 10.1038/nature01883

[ele70333-bib-0083] Wang, X.‐W. , and Y.‐Y. Liu . 2023. “Origins of Scaling Laws in Microbial Dynamics.” Physical Review Research 5: 013004.

[ele70333-bib-0084] West, R. , and N. M. Shnerb . 2022. “Quantitative Characteristics of Stabilizing and Equalizing Mechanisms.” American Naturalist 200: E160. 10.1086/720665 36150202

[ele70333-bib-0085] Yenni, G. , P. B. Adler , and S. K. M. Ernest . 2017. “Do Persistent Rare Species Experience Stronger Negative Frequency Dependence Than Common Species?” Global Ecology and Biogeography 26: 513.

