# Supplementary material for: Fluctuating Growth Rates Link Turnover and Unevenness in Species‐Rich Communities
Source: Ecol Lett. 2026 Feb 12;29(2):e70333. doi: 10.1111/ele.70333 (PMC12895236; doi:10.1111/ele.70333)
Supplement: Supplementary file 1 — Data S1: ele70333‐sup‐0001‐DataS1.pdf. [file ELE-29-0-s001.pdf]

Supplementary Information
Fluctuating growth rates link turnover and unevenness in species-rich communities

Emil Mallmin[1,*], Arne Traulsen[1], and Silvia De Monte[1,2]

[1]Max Planck Institute for Evolutionary Biology, Plön, Germany
[2]Institut de Biologie de l'ENS (IBENS), Département de Biologie, Ecole Normale Supérieure, CNRS, INSERM, Université PSL, 75005 Paris, France
[*]mallmin@evolbio.mpg.de

Wednesday 24th September, 2025

Supplementary Tables

S1 Table of variables, parameters, and abbreviations. 2

Supplementary Figures

S1 Goodness of fit for the focal species model SAD prediction given simulated noise statistics. 1
S2 Systematic change in SAD shape by traversing the Buffering-Stabilization parameter plane. 3
S3 Time-evolution of the SAD with or without coexistence mechanisms 4
S4 Turnover measured by Bray-Curtis decay corresponding to the panels in Figure S2. 5
S5 Turnover measured by Bray-Curtis decay corresponding to Main Text Figure 6. 5
S6 Distribution of mean abundances for the panels in Main Text Figure 6. 6

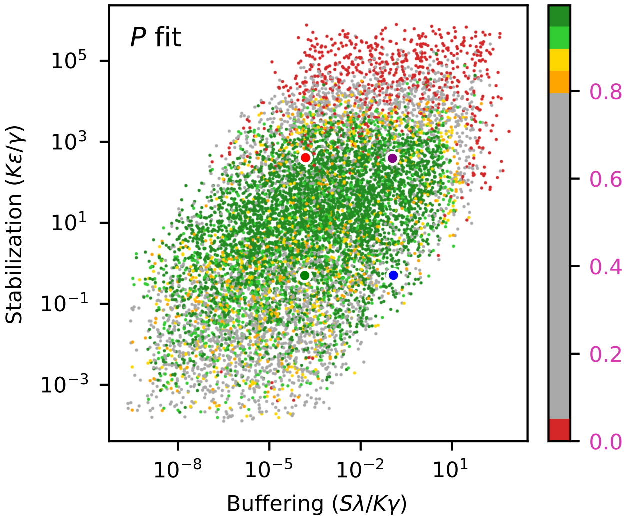

Figure S1: Goodness of fit for the focal species model SAD prediction given simulated noise statistics. For each simulation we have produced $P(n)$ according to Main Text Eq. (17), with the parameters ν, a, b obtained from Main Text Eq. (18) and (19) using the values of $\overline{N}$ and $\mathrm{Var}[N]$ from the simulations. The goodness of fit of $P(n)$ with the time-averaged FAD (from 0 (no match) to 1 (perfect match)) is measured by (one minus) the Kolmogorov-Smirnov distance of the distributions: $1 - \sup_n |F_P(n) - F_{\mathrm{FAD}}(n)|$.

**Dynamical variables**		
$n_i(t)$	abundance of species i ($n_{i,\alpha}(t)$ for patch α)	
$r_i(t)$	intrinsic growth rate or environmental fitness—*fitness*, for short—of species i	
Derived quantities		
$N(t)$	$:= \sum_i n_i(t)$; total abundance	
$p_i(t)$	$:= n_i(t)/N(t)$; relative abundance	
$\rho(t)$	$:= \sum_i r_i(t)p_i(t)$; community-averaged fitness	
$S_{\text{eff}}(t)$	$:= [\sum_i p_i^2(t)]^{-1}$; effective species richness, i.a. Simpson's (reciprocal) diversity index	
**Base parameters**		
The fundamental parameters that fully determine the model (note that μ and σ_r^2 are redundant):		
S	number of species	—range 100–1000
μ	heterospecific interaction rate	—implied by r^*, K; always 1
ε	excess self-regulation	—range 0.01–100 or zero
r^*	fitness mean value	—fixed to 1 by non-dimensionalization
K	$:= r^*/\mu$; carrying capacity	—fixed to 1 by non-dimensionalization
λ	immigration rate	—range 10^{-10}–10^{-4} or zero
τ	autocorrelation time; $\text{Corr}[r_i(t), r_j(t')] = \delta_{ij}e^{-\lvert t-t'\rvert/\tau}$	—default value 10; range 0.01–100
σ_r	std of fitness fluctuations	—implied by τ, γ; default value 0.05
γ	$:= 2\sigma_r^2\tau$; rate of stochastic exclusion; env. noise amplitude squared	—default value 0.05; range 10^{-4}–100
n_{ext}	extinction cutoff	—range 10^{-12}–10^{-3} or absent (zero)
Instead of λ in the multi-patch model:		
M	number of patches	—range 1–80
$d_{\beta\alpha}$	rate of immigration from patch α to β	—0.001 or $0.01/M$
In the scenario without time-average neutrality:		
r_i^*	mean fitness of species i; drawn uniformly from $r^* \pm \delta r^*$	—maximal range 0.5–1.5
**Derived parameters**		
Compound parameters defining the Buffering–Stabilization plane		
B	$:= S\lambda/K\gamma$; Buffering	
Σ	$:= K\varepsilon/\gamma$; Stabilization	
'Effective parameters' determined implicitly by the community dynamics as the parameters of the OUP approximation of $r_i(t) - \mu N(t)$		
r^*_{eff}	effective mean fitness	
$\sigma_{r_{\text{eff}}}$	effective fitness std	
τ_{eff}	effective fitness autocorrelation time	
Parameters of the GIG distribution describing the SAD and FAD under TAN		
a	$:= \gamma_{\text{eff}}/2\varepsilon$; characteristic abundance of right bend	
b	$:= 2\lambda/\gamma_{\text{eff}}$; characteristic abundance of left bend	
ν	$:= 1 - 2r^*_{\text{eff}}/\gamma_{\text{eff}}$; exponent of the (inverse) power law section	
**Abbreviations**		
TAN	Time-average neutrality; species have identical expected fitness	
SAD	Species–abundance distribution; fraction of species vs abundance class	
FAD	Frequency–abundance distribution; fraction of time spent in abundance class by one particular species	
GIG	generalized inverse Gaussian (distribution); a three-parameter 'bent' power law	

Table S1: Table of variables, parameters, and abbreviations. The parameter values used in simulation are stated in the corresponding figure captions. Here, we give an indication of the default values and/or ranges considered.

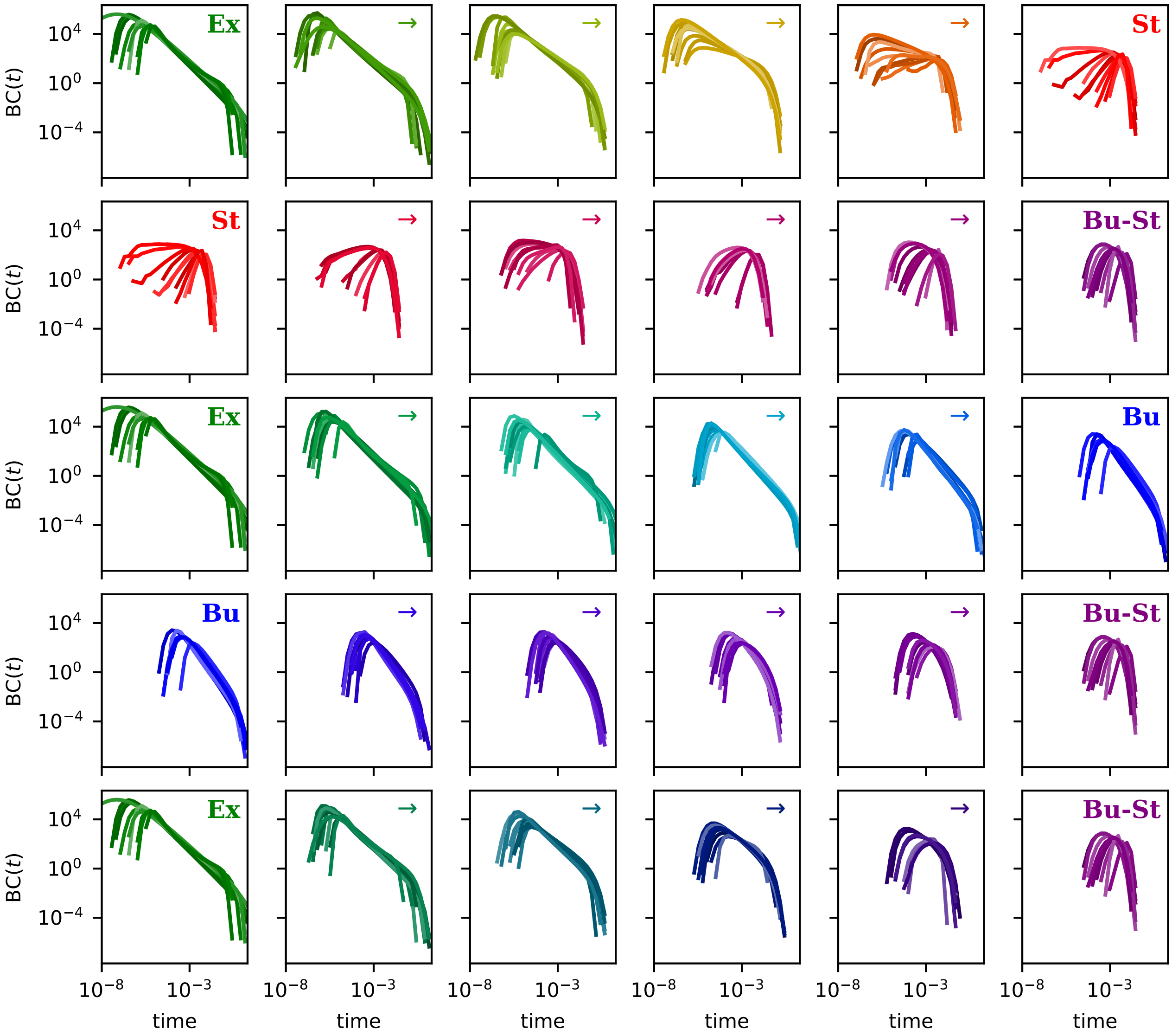

Figure S2: Systematic change in SAD shape by traversing the Buffering-Stabilization parameter plane. (With reference to Main Text Figure 5.) Here we show the distribution sets that lie on a straight line between the reference cases, e.g. between Ex and St, in the upper row; Ex and Bu-St in the second row; and so on. The variation in line colors within a panel are just a guide for the eye.

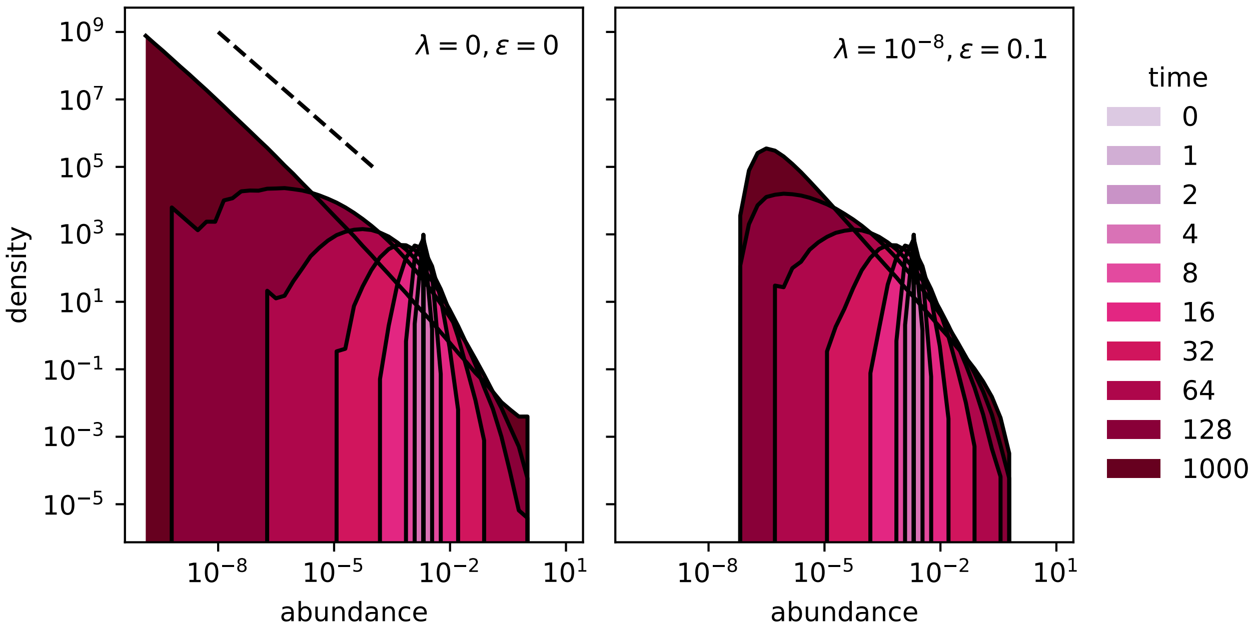

Figure S3: Time-evolution of the SAD with or without coexistence mechanisms. Starting from an even initial condition, we track the evolution of the SAD averaged over 1000 realizations. In the left panel, there is no immigration and no additional self-suppression, in contrast to the right panel. Early on, both scenarios give similar distributions, until the bounds in the latter scenario restrict the expansion of the distribution; for the former scenario, the power-law section extends ever into lower abundance decades with time, seemingly approaching an exponent of 1 (dashed line). Here, $S = 500, r^* = 1, \sigma_r = 0.05, \tau = 10$.

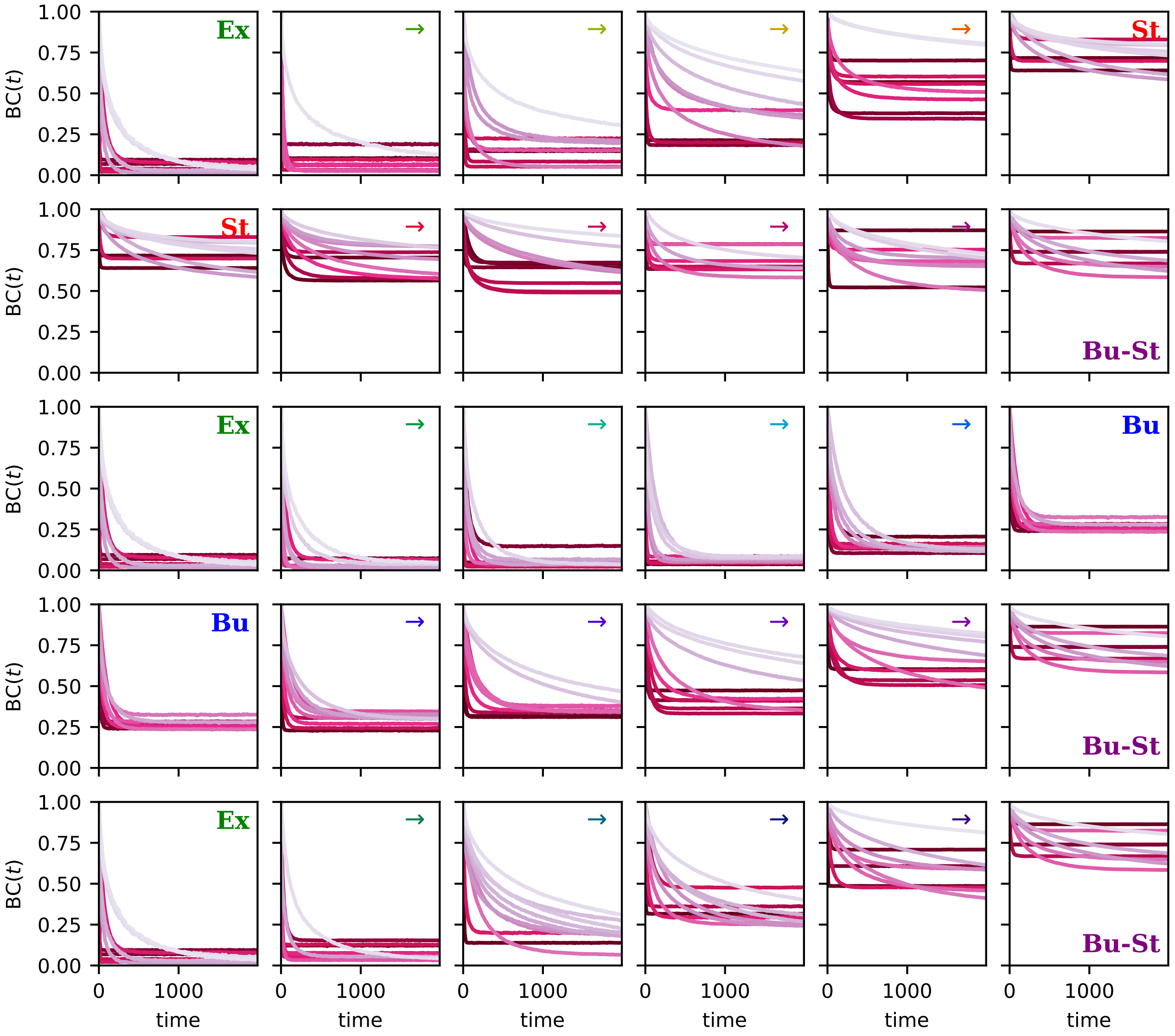

Figure S4: Turnover measured by Bray-Curtis decay corresponding to the panels in Figure S2. The color of the line reflects the value of $\log_{10}\gamma$, normalized separately for each panel—light colors for small γ, dark color for large. Note that small γ gives slower decay, and that narrow SADs are associated with high limit of the BC.

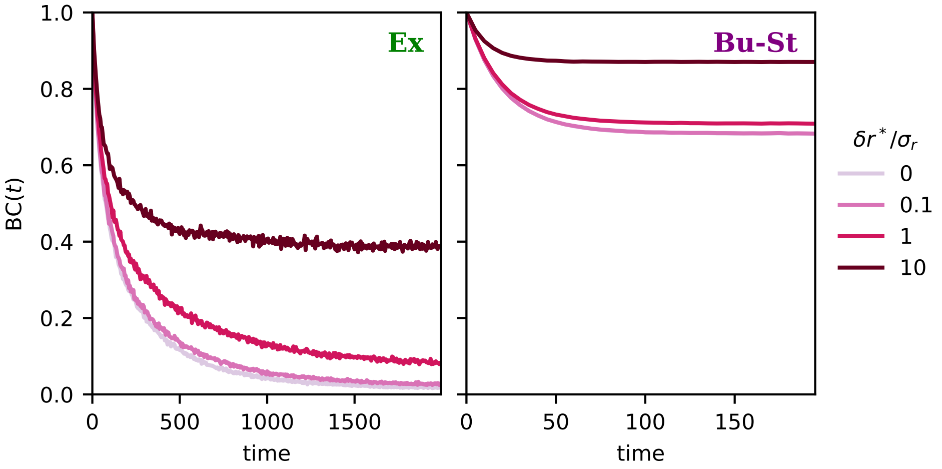

Figure S5: Turnover measured by Bray-Curtis decay corresponding to Main Text Figure 6. For each scenario (row) of Main Text Figure 6, each of the four panels with different $\delta r^*/\sigma_r$ corresponds to one line. Note the 10x difference in timescale between the two scenarios.

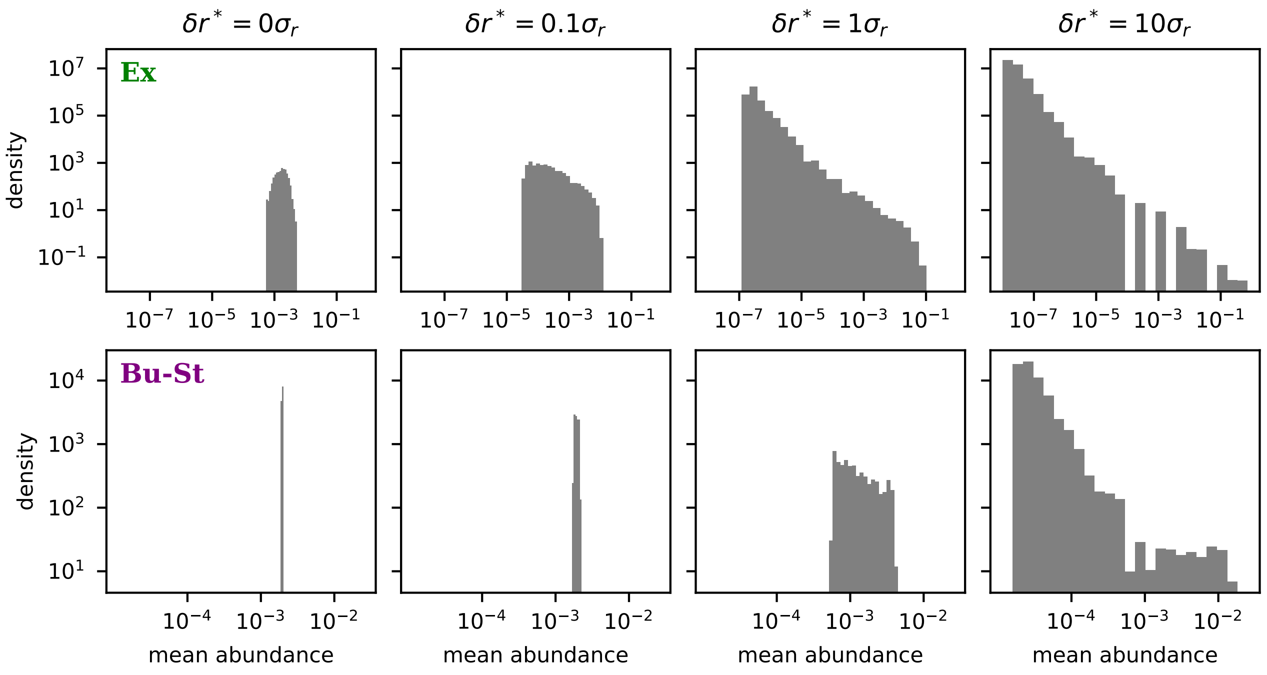

Figure S6: Distribution of mean abundances for the panels in Main Text Figure 6. Breaking time-averaged neutrality produces a distribution of species means. Note that the panels of the first column are TAN, so theoretically all species would have the same mean if the sampling time window was infinite. Note also the difference in scale of the horizontal axis of the two rows.
